# Crossroads between peripheral atherosclerosis, western-type diet and skeletal muscle pathophysiology: emphasis on apolipoprotein E deficiency and peripheral arterial disease

**DOI:** 10.1186/s12929-017-0346-8

**Published:** 2017-07-08

**Authors:** Peggy Sfyri, Antonios Matsakas

**Affiliations:** Molecular Physiology Laboratory, Centre for Atherothrombotic & Metabolic Disease, Hull York Medical School, University of Hull, Cottingham Road, Hull, HU6 7RX United Kingdom

**Keywords:** Atherosclerosis, Apolipoprotein E, NADPH oxidases, Oxidative stress, Peripheral arterial disease, Skeletal muscle

## Abstract

Atherosclerosis is a chronic inflammatory process that, in the presence of hyperlipidaemia, promotes the formation of atheromatous plaques in large vessels of the cardiovascular system. It also affects peripheral arteries with major implications for a number of other non-vascular tissues such as the skeletal muscle, the liver and the kidney. The aim of this review is to critically discuss and assimilate current knowledge on the impact of peripheral atherosclerosis and its implications on skeletal muscle homeostasis. Accumulating data suggests that manifestations of peripheral atherosclerosis in skeletal muscle originates in a combination of increased i)-oxidative stress, ii)-inflammation, iii)-mitochondrial deficits, iv)-altered myofibre morphology and fibrosis, v)-chronic ischemia followed by impaired oxygen supply, vi)-reduced capillary density, vii)- proteolysis and viii)-apoptosis. These structural, biochemical and pathophysiological alterations impact on skeletal muscle metabolic and physiologic homeostasis and its capacity to generate force, which further affects the individual’s quality of life. Particular emphasis is given on two major areas representing basic and applied science respectively: a)-the abundant evidence from a well-recognised atherogenic model; the Apolipoprotein E deficient mouse and the role of a western-type diet and b)-on skeletal myopathy and oxidative stress-induced myofibre damage from human studies on peripheral arterial disease. A significant source of reactive oxygen species production and oxidative stress in cardiovascular disease is the family of NADPH oxidases that contribute to several pathologies. Finally, strategies targeting NADPH oxidases in skeletal muscle in an attempt to attenuate cellular oxidative stress are highlighted, providing a better understanding of the crossroads between peripheral atherosclerosis and skeletal muscle pathophysiology.

## Background

Hyperlipidaemia – defined as increased levels of lipids and/or lipoproteins in blood – is a major risk factor of atherosclerosis and cardiovascular disease as well as an independent risk factor for peripheral arterial disease (PAD) [1, 2]. Atherosclerosis exhibits systemic manifestations not only in the large vessels of the heart (e.g. coronary arteries) but also affects peripheral vessels [3, 4]. Atherosclerosis in peripheral arteries has a considerable impact on skeletal muscle pathophysiology. PAD patients exhibit a myopathy in the affected limbs, as a result of increased oxidative damage and mitochondrial dysfunction. As the disease progresses patients show myofibre degeneration, fatty acid deposition, fibrosis and decreased capillarisation [5–9]. These features are followed by intermittent claudication, reduced exercise tolerance and ambulation that compromise the quality of life of PAD patients [10–14].

The transport of triglycerides, chylomicrons, chylomicron remnants and other lipids through the lymphatic and circulatory system is regulated by apolipoproteins. These proteins bind with lipids and phospholipids to form lipoprotein complexes in a variety of sizes according to the relative amounts of lipids and proteins they incorporate [15]. Lipoprotein complexes are composed mainly of triglycerides, cholesteryl esters and a small portion of lipid soluble vitamins, while their surface is covered by phospholipids, unesterified cholesterol and apolipoproteins [16]. In addition, apolipoproteins act as cofactors or activators of enzymes that are involved in cholesterol metabolism, by mediating cholesterol esterification and lipid catabolism [17]. As a result of their role in lipoprotein metabolism, apolipoproteins are important contributors to plasma lipid homeostasis and are used in the prognosis of coronary artery disease [18]. However, mutations of apolipoprotein genes B (*APOB*) or E (*APOE*) lead to familial hypercholesterolaemia or dysbetalipoproteinaemia; diseases that are associated with premature atherosclerosis [19].

Apolipoprotein E (APOE) regulates blood lipid and lipoprotein levels in multiple ways, including acting as a ligand for low-density lipoprotein receptor (LDLR), the transfer of lipoproteins to the liver, the formation of chylomicrons and very low-density lipoproteins (VLDL) as well as affecting the activities of hepatic and lipoprotein lipases [20, 21]. However, its functions extend far beyond the regulation of lipid metabolism. Recent evidence suggests that APOE plays a role in normal brain function and a number of cardiovascular, neurodegenerative (such as Alzheimer disease, Parkinson’s disease and multiple sclerosis) as well as metabolic (e.g. type 2 diabetes mellitus) diseases [20]. ApoE deficiency in mice has been also shown to result in spontanenous atherosclerotic plaque formation also seen in humans with severe hypercholesterolaemia (reviewed in [22]). In turn, atherosclerosis is a chronic disorder that is associated with cellular inflammation and oxidative stress. Reactive oxygen species (ROS) elicit cellular damage by initiating chemical chain reactions such as lipid peroxidation, protein oxidation as well as DNA damage [22, 23]. Increased ROS production is one of the major contributors of atherosclerosis that leads to endothelial dysfunction. Nicotinamide adenine dinucleotide phosphate (NADPH) oxidases (Noxs) have received a lot of attention due to their contribution in ROS production and nicotinamide adenine dinucleotide phosphate oxidase 2 (Nox2) in particular has been shown to be involved in atherosclerotic lesions [24–26].

The aim of this review is to critically discuss and assimilate current knowledge on the impact of peripheral atherosclerosis and its implications on skeletal muscle homeostasis. Accumulating data suggest that manifestations of peripheral atherosclerosis in skeletal muscle originate in a combination of increased i) oxidative stress, ii) inflammation, iii) mitochondriopathy, iv) altered myofibre morphology and fibrosis, v) chronic ischemia followed by impaired oxygen supply, vi) reduced capillary density and vii) apoptosis [6, 7, 9, 27]. These biochemical and pathophysiological alterations impact on skeletal muscle’s metabolic and physiologic homeostasis and in turn on its capacity to generate force which may further affect the individual’s quality of life. Particular emphasis is given on i) the abundant evidence from the ApoE deficient mouse model and the role of western-type of diet and ii) on skeletal myopathy and oxidative stress-induced myofibre damage from human studies. At last, recent findings from experimental models of atherosclerosis regarding the role of NADPH oxidases in atherothrombotic disease are discussed in detail.

## Biological functions of apolipoprotein E

Apolipoprotein E is synthesized by several tissues, primarily the liver, brain, adrenal glands, adipose tissue, kidney and macrophages [28–30]. APOE associates with triglyceride-rich proteins such as VLDL and chylomicrons as well as a subset of high-density lipoproteins (HDL) [31, 32]. It also serves as a ligand for the LDL receptor, LDL receptor related protein 1, APOE receptor 2 and heparan sulfate proteoglycans [33–35]. The human *APOE* gene is located on chromosome 19 and is comprised of four exons and three introns [31]. APOE is synthesized as a 319 amino acid propeptide and undergoes mucin-O-type glycosylation in the Golgi apparatus, giving rise to the mature APOE protein consisting of 299 amino acids with a molecular weight of ~34.5 kDa [31, 34, 36]. The *APOE* gene has two polymorphisms at a single locus that lead to three *APOE* alleles, *ɛ2 (APOE2)*, *ɛ3 (APOE3)* and *ɛ4 (APOE4)* [34]. The different combination of the above alleles gives rise to three heterozygous and three homozygous phenotypes [37]. APOE3 is the most prevalent isoform and is considered to be the allele that all other variants have derived from [34]. APOE2 and APOE4 are associated with several inflammatory diseases [38]. APOE2, in particular, correlates with the genetic lipid disorder type III hyperlipoproteinaemia and high plasma triglyceride levels [39]. APOE4 on the other hand, correlates with increased plasma levels of LDL, thus contributing to a higher risk for developing cardiovascular disease and is also associated with the initiation of Alzheimer’s disease [37–40]. The key role of APOE in atherosclerosis has been reviewed previously [21, 28, 32, 34, 41, 42].

APOE has a major anti-atherogenic function, as it regulates plasma homeostasis by promoting the clearance of lipoproteins, chylomicrons and their remnants, induces VLDL secretion from the liver as well as the reverse cholesterol efflux of VLDL and chylomicron remnants from the liver and macrophages [28, 37, 42]. The key role of APOE in lipoprotein metabolism is brought about by the activation of enzymes that are essential for lipoprotein breakdown. In addition to the lipoprotein metabolism, APOE exhibits anti-atherogenic activity by inhibiting platelet aggregation in the vessel wall and induces proliferation of T lymphocytes, endothelial cells and vascular smooth muscle cells [34]. Apart from its anti-atherogenic role and regulation of lipoprotein metabolism, APOE has been shown to be neuroprotective, as well as acting as an antioxidant and immunomodulator by suppressing type I inflammatory response and inducing the M2 anti-inflammatory pathway in macrophages [31, 33, 34, 43]. Furthermore, accumulating data report that APOE is involved in adipogenesis by inducing the assembly of triglycerides in the adipocytes [39]. Therefore, ApoE deficiency has been used extensively in experimental studies of atherosclerosis.

## Manifestations of ApoE deficiency in the cardiovascular system

APOE deficiency in humans is a rare phenomenon but has a broad range of phenotypes spanning from mild hypercholesterolaemia to severe xanthomatosis, hyperlipoproteinaemia and development of premature atherosclerosis [37, 41, 44, 45]. Similarly, ApoE deficiency in mice (ApoE^-/-^) leads to hyperlipidaemia and spontaneous development of atherosclerotic lesions from the age of 10 weeks, designating the ApoE^-/-^ mouse model as one of the best established experimental tools to study atherosclerosis [46, 47]. Consequently, a plethora of studies conducted in ApoE^-/-^ mice has addressed the impact of the initiation and progression of atherogenesis on the vascular system [48–51].

The majority of cholesterol in the ApoE^-/-^ mice is distributed in VLDL and intermediate density lipoproteins (IDL)/LDL particles [52, 53]. It is well known that plasma cholesterol and particularly VLDL and IDL remnants are significantly elevated in ApoE^-/-^ mice as summarised in Table 1 (e.g. [47, 49]). Compared to wild type mice, total plasma cholesterol levels increase by 3- to 7-fold – depending on the study and the age of mice–while plasma triglycerides show an up to twofold increase [47, 54–56]. LDL increases by 4– to 14–fold and VLDL and IDL remnants by 10– to 18–fold [47]. There are conflicting data with regards to HDL levels that have been shown to decrease, remain unchanged or even increase [45, 48, 55, 57, 58]. Tani et al. reported that ApoE^-/-^ mice not only have lower levels of HDL cholesterol, but the HDL particles are significantly altered in their composition [45].

**Table 1:** Overview of blood lipid and lipoprotein changes in response to ApoE deficiency and/or western-type diet

	ApoE^-/-^ ND vs. WT ND	ApoE^-/-^ WD vs. WT WD
Total Cholesterol	↑ 3–7fold [48, 52, 54, 287]	↑ 2–18fold [48, 52, 54, 99, 100, 287]
VLDL/IDL	↑ 10fold [52, 54, 79, 95]	↑ 10–30fold [47, 52, 54, 95]
LDL	↑ 4-14fold [58, 79, 95]	↑ 3–14fold [47, 54, 95]
TGs	↑ 0–4fold [48, 52, 54, 58, 100, 287]	↑ 0–2fold [48, 52, 54, 58, 100, 287]
HDL	↓ 0–1.7fold [15, 48, 58, 79, 95]	↓ 0–2fold or ↑3fold [48, 52, 95, 98, 103]

Abbreviations: *HDL* High-density lipoprotein, *LDL* Low-density lipoprotein, *ND* Normal chow diet, *VLDL/IDL* Very low-density lipoprotein/Intermediate density lipoprotein, *TGs* Triglycerides, *WD* Western-type diet, ↑Increase, ↓Decrease

Due to their hyperlipidaemic profile, ApoE^-/-^ mice develop atherosclerotic lesions at a young age. Monocytes begin to adhere to the vascular wall at the age of 6–8 weeks and approximately after 2 weeks; foam cells and lesions are observed leading to atherosclerotic plaques [22, 59, 60]. Plaque formation is apparent initially in the proximal aorta and progressively expands throughout the aorta showing diversity in the size, number and complexity [61]. At the onset of atherosclerotic lesion, inflammation is augmented in the aorta [22]. Specifically, vascular cell adhesion molecule 1 expression (VCAM1) from aortic endothelial cells is increased and a significant upregulation of genes that are involved in inflammation and proteolysis is observed from the age of 12 weeks [61, 62]. Apart from increased inflammation in the aorta, there is evidence of oxidative stress, impaired mitochondrial respiration and mitochondrial DNA deletion that increases by 3–fold at a later age [63–67]. Nonetheless, antioxidant protein expression in the aorta is not altered at the age of 10 weeks [64].

As atherosclerotic lesions advance with age, plasma levels of VLDL and LDL either increase or remain unchanged [49, 68]. Peritoneal macrophages that form lipid–laden foam cells exhibit decreased expression of ATP–binding cassette transporter A1 and Scavenger receptor type B1 –both contributors of anti-atherogenic properties in macrophages–and increased expression of Cluster of Differentiation 36–a receptor with pro-atherogenic functions and thus may further contribute to lesion progression [49]. Along with the lesion progression in size and composition, the endothelium is severely damaged with increased leakage, loss of tight junctions of endothelial cells and increased repair, while a decrease in endothelial progenitor cells that are involved in vascular repair is observed [69, 70]. At 13 months of age, ApoE^-/-^ mice exhibit a 61% occlusion of the lumen and decreased endothelial-dependent relaxation to acetylcholine, an increase in stiffness of conduit arteries leading to elastic laminar fragmentation, while endothelium independent relaxation remains intact [68, 71–74]. In contrast to humans, the impairment in endothelium dependent vasodilation in ApoE^-/-^ mice appears to be focal rather than systemic [47, 75]. Additionally, the extend of mitochondrial DNA deletion in the aorta is greater as shown by decreased mRNA and protein levels of 8-oxyguanine glycosylase – a DNA repair enzyme [66].

At 17–18 months of age, lumen occlusion can reach 90% and lesions are found mostly in the aortic arch, aortic root and in the proximal and distal segments of thoracic aorta, while small lesions appear in the central thoracic aorta [59, 72, 76]. Moreover, lipid deposition is further augmented and vascular senescence appears mainly in the aortic arch, a site with turbulent blood flow, and as a compensatory mechanism for maintaining the diameter of the vessel positive remodelling is observed in the aorta [56]. Hence, atherosclerosis in ApoE^-/-^ mice advances in an age-dependent manner and lesion composition and progression is very much similar in humans [47, 77]. It is worth mentioning that even though there is a significant similarity in the atherosclerotic process between ApoE^-/-^ mice and humans, ApoE^-/-^ mice do not develop lesions in the coronary arteries as humans [59, 61]. Many studies have shown an increased heart-to-body mass ratio (an index of cardiac hypertrophy), left ventricular hypertrophy and increased diameter of the posterior wall from the age of 13 weeks that progress with age [73, 78–80]. In parallel, 10 month-old ApoE^-/-^ mice exhibit dilated cardiomyopathy characterised by impaired left ventricular function and increased fibrosis of the myocardium and endocardium [68]. Although ApoE^-/-^ deficiency in combination with ageing and diet seem to impact on cardiac morphology [22], there is a number of studies that failed to report cardiac phenotypic changes [56, 81, 82], suggesting that other variables may play a role in the multifactorial development of a cardiac phenotype.

The progression of atherosclerosis may be regulated by sex [47]. Some evidence suggests that there is a significant difference in total plasma cholesterol and triglyceride levels between male and female ApoE^-/-^ mice of the same age and diet, with male mice displaying higher levels [54, 56, 77]. However, this finding has not been confirmed by others [83, 84]. Furthermore, many groups have reported that lesion surface area in the aorta of male mice is greater than in females and Chiba et al. (2011) suggest that the difference might be due to increased activity of neutral cholesterol ester hydrolase by estradiol in the aorta and peritoneal macrophages [49, 56, 83, 85]. Studies in gonadectomised female ApoE^-/-^ mice showed that the lesion burden in the aortic sinus and abdominal aorta was greater that in control ApoE^-/-^ mice, while estradiol 2 treatment of gonadectomised male and female ApoE^-/-^ mice led to a 50% decrease of lesion area throughout the aorta [86, 87]. On the contrary, other studies report that lesion coverage is either greater in female than in male mice or no correlation is found based on sex [59, 77, 88, 89]. In a recent study using western-type diet, female mice had greater endothelial dysfunction in the coronary arteries than male mice [84]. Taken together, the above data suggest that although the male sex can affect the progression of atherosclerosis there may be other contributing variables such as diet that play a role in atherogenesis and additional research is warranted.

## High-fat and western-type diets accelerate atherosclerosis in ApoE deficiency

High-fat diets are usually composed of 15–36% w/w fat which corresponds to 34–60% kcal from this source containing mostly saturated fats [90–95]. However, several studies use the term “western diet” in an attempt to draw parallels with the dietary composition of modern societies, which are rich in fat and cholesterol. A western-type diet is typically composed of 21% w/w fat that corresponds to 40–45% kcal from fat supplemented with 0.15–1.25% cholesterol [53, 96]. High-fat and western-type diets have both been used in ApoE^-/-^ mice and abundant evidence suggests that such dietary challenges result in aggravation of the atherosclerotic phenotype [61, 95, 97].

One to eight weeks on a western-type diet increases total plasma cholesterol levels by 3- to 7-fold and atherosclerotic lesions in the aortic sinus are evident from the fourth week in young ApoE^-/-^ mice [48, 98]. Western-type diet administration for larger periods such as 24 weeks results in total cholesterol levels of 1200–1400 mg/dL, representing a 12- to 18-fold change compared to wild type mice or a 5-fold change compared to ApoE^-/-^ mice kept on a chow diet, although absolute levels of total cholesterol vary amongst studies as shown in Table 1 [48, 98–100]. Aortic lesion coverage after 5 weeks of a western-type diet is minimal, approximately 1.8%, and reaches 5.5% after 12 weeks [57]. However, the abdominal aorta lesion coverage after 14 weeks of western-type diet reaches 19% and after 24 weeks the plaque surface area covers about 60% of the aorta [50, 98]. Atherosclerosis is considered an inflammatory process and expression of pro-inflammatory molecules such as intercellular adhesion molecule 1 (iCAM–1) and C-C motif chemokine receptor 2 protein and mRNA expression of interleukin 6, interleukin 17 and inducible nitric oxide synthase in the aorta appear to be augmented from the second week of western-type diet, in the absence of changes in plasma pro-inflammatory cytokines interleukin 6 and 10 and tumour necrosis factor α (TNFα) [48, 50, 98]. Other inflammatory markers, such as interleukins 1α, 1β and VCAM-1, are also elevated in the aorta [101, 102]. However, others have not observed alterations in iCAM–1 levels in endothelial cells before 20 weeks of a western-type diet [61]. Apart from macrophage infiltration, Type 1, Type 2 and regulatory T-helper lymphocytes cells are also recruited in the aorta, with T-helper Type 2 (Th2) cells reducing after 10 weeks of a western-type diet whereas T-helper Type 1 (Th1) lymphocytes and regulatory T-helper cells remain in the lesions [50]. Th1 lymphocytes notably exhibit pro-inflammatory properties whereas Th2 and regulatory T helper cells are anti-inflammatory [50]. Further to the increase of Th1 cells, upregulation of T-helper Type 17 (Th17) cells and subsequent expression of interleukin 17 that advocates atherosclerosis are apparent in the aorta and as the duration of a western-type diet advances, Th17 and Th1 cells accrete in splenocytes, suggesting that there is a systemic upregulation of T helper lymphocytes [98, 103].

Along with increased inflammation, increased oxidative stress is also evident. By 7 weeks of western-type diet, reactive oxygen species production increases by 2-fold in the aorta and after 8 weeks there is a 4-fold increase in the media of the aorta and a 3-fold increase in the perivascular fat of the ascending aorta [48, 57]. This increase has been mainly attributed to enzymes that generate ROS. Nox2 expression is increased by 3-fold in macrophages and endothelial cells and xanthine oxidase expression is augmented in macrophages, endothelial cells and smooth vascular cells [57, 102]. After 12 weeks on the western-type diet there are elevated markers of oxidative stress not only in the aorta, but also in plasma [101]. Moreover, hypoxia is considered to contribute in plaque progression possibly through the induction of several genes that are involved in inflammation, redox homeostasis, apoptosis and neovascularisation. Evidence is based on the expression of hypoxia inducible factor 1α (HIF1α) and its downstream targets vascular endothelial growth factor (VEGF) and glucose transporter 1 specifically in the central regions of lesions, where oxygen supply is attenuated after a 16- or 30-week western-type diet challenge [97, 104].

Endothelial dysfunction is evident from the fifth week of western-type diet and in particular, endothelium-dependent relaxation to acetylcholine is impaired in the proximal descending aorta whereas it remains unaltered in the thoracic aorta [105, 106]. Endothelial nitric oxide synthase (eNOS) and protein kinase B phosphorylation levels are decreased in the aorta of ApoE^-/-^ mice after 16 weeks of western-type diet when compared to the wild type mice on a chow diet [107]. Conversely, ApoE^-/-^ mice fed a western-type diet for 20 weeks have increased eNOS protein expression in aortas when compared to wild type fed the same diet, potentially as a compensatory mechanism against the increased expression of Nox2 that reduces nitric oxide bioavailability [108]. Endothelium-dependent relaxation to acetylcholine is further impaired after 30 weeks of western-type diet and the impairment is possibly due to decreased Ca^2+^ dependent eNOS activity as judged by decreased basal levels of cyclic guanosine monophosphate [109]. The wall of the aorta increases in thickness and stiffness as the duration of the western-type diet extends, as shown from in situ extension-inflation tests [97]. In resistance vessels, such as mesenteric arteries, endothelium-dependent and endothelium independent vasodilation are impaired and there is an increase in wall thickness and cross sectional area possibly because of increased expression of vasoconstrictors such as endothelin 1 (ET-1) [109, 110]. Similarly, in carotid arteries after 12 months of a western-type diet, positive remodelling is observed that is inadequate to compensate for the decrease of lumen diameter and over 70% stenosis is apparent, followed by a decline in vasodilation to acetylcholine and nitric oxide [111].

In addition to endothelial dysfunction, increased inflammation and oxidative stress, there is evidence that ApoE deficiency may impact on cardiac muscle per se. Standard chow diet supplemented with 0.15% w/w cholesterol for 18 months induces cardiac hypertrophy followed by impaired cardiac function which is characterised by a reduced ejection fraction and a reduced maximum rate of change of left ventricular pressure [112]. In turn, a western-type diet to 40 week old mice results in increased cardiomyocyte size and subsequent cardiac hypertrophy, an age-dependent increase in left ventricular mass, followed by increased collagen volume and myocardial fibrosis [60, 113, 114]. However, other studies failed to detect any changes in the heart-to-body weight ratio after 30 weeks of western-type diet [84, 98]. This discrepancy may be due to the diet composition, atherogenicity of the diet (i.e. cholesterol amount) as well as the experimental diversity regarding the animal age.

Apart from the ApoE^-/-^ mice, LDL receptor deficient (Ldlr^-/-^) mice represent another model of systemic atherosclerosis that shows mechanistic differences in the development of atherosclerosis reviewed elsewhere [54, 93]. In brief, Ldlr^-/-^ mice on a chow diet have also increased total plasma cholesterol but lesion formation is rather observed in aged animals and is limited to the aortic root, whereas in ApoE^-/-^  mice lesions form at 8–10 weeks of age and subsequently develop throughout the aorta. When ApoE^-/-^ mice are fed a western-type diet total plasma cholesterol is further augmented and lesions form at an earlier age (i.e. 4–6 weeks old) and progress more rapidly [93, 115, 116]. Conversely, Ldlr^-/-^ mice fed an atherogenic diet for 12 weeks, develop lesions in the aortic root and increase thereafter robustly extending to the thoracic and abdominal regions of the aorta [115, 117]. Another difference between the two atherosclerotic models is that ApoE^-/-^ mice subjected to either a chow- or an atherogenic- diet carry most of their cholesterol in the apoB48-containing VLDL particles, whereas Ldlr^-/-^ mice on a chow diet have predominantly apoB100-containing LDL particles, a feature that is more relevant to human pathogenesis of atherosclerosis [54, 93]. However, when challenged with an atherogenic diet, Ldlr^-/-^ mice exhibit increased LDL as well as VLDL particles [93, 118]. Furthermore, ApoE is a lipoprotein that not only contributes to lipid homeostasis but has several other functions (such as immunomodulator and antioxidant) as discussed in a previous section. Therefore, findings in the ApoE^-/-^ mice may be attributed not only to the plasma lipid levels but also to the loss of the antiatherogenic functions of ApoE [93, 119].

Beyond the well-documented evidence on the detrimental effects of atherogenic diets with regards to the cardiovascular system and progression of atherosclerosis, there has recently been an expansion of interest on new areas that will be discussed below. Emphasis is given on i) the manifestations of atherosclerosis in peripheral tissues such as skeletal muscle and ii) the role of Noxs in vascular disease and the subsequent potential emerging from strategies to attenuate vascular oxidative stress by targeting NADPH oxidases.

## Manifestations of peripheral atherosclerosis in human skeletal muscle

**Fig. 1:**
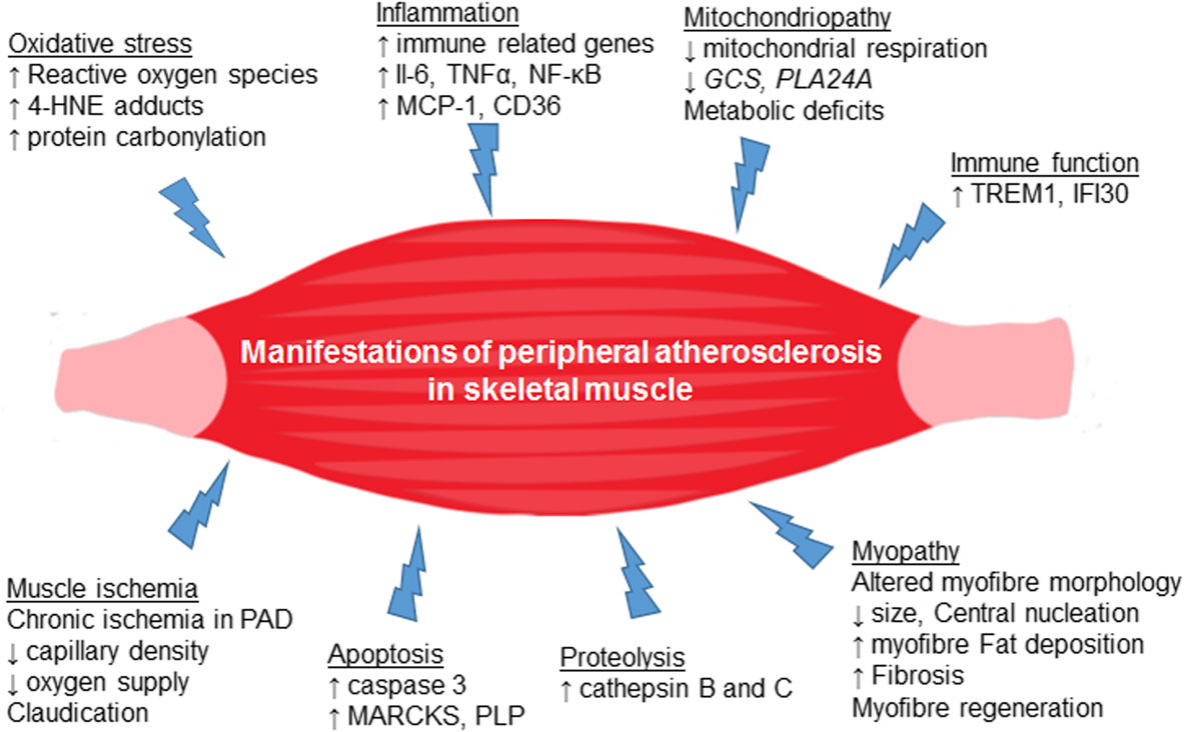
Manifestations of peripheral atherosclerosis in skeletal muscle structure, function and metabolic homeostasis. *﻿﻿4-HNE adducts*: 4-hydroxy-2-nonenal adducts;﻿ *CD36*: Cluster of differentiation 36; *GCS*: glycine cleavage system protein H; *IFI30*: Gamma-interferon-inducible lysosomal thiol reductase, *IL-6*: Interleukin 6; *MARCKS*: myristoylated alanine-rich C kinase substrate; *MCP1*: monocyte chemotactic protein 1; *NF-κB*: Nuclear factor kappa-light-chain-enhancer of activated B cells; *PLA24A*: phospholipase A2 group IVA; *PLP*: phospholipid transfer protein; *TNFα*: tumour necrosis factor α; *TREM1*: Triggering receptor expressed on myeloid cells 1

Skeletal muscle constitutes one of the largest tissues in the human body comprising of almost 40% total body weight [120]. Skeletal muscle is a very dynamic and adaptable tissue that accounts for up to 40% of the metabolic rate at rest in humans and provides for 30–50% of protein turnover for the whole body [121]. The main role of skeletal muscle is to generate force and movement which is accomplished by turning chemical energy (ATP) into mechanical energy. Apart from the production of mechanical energy, muscle exhibits multiple metabolic functions [122]. It serves as a reservoir of amino acids that are used by other tissues, generates body heat, has a high capacity of oxidative metabolism and represents the major peripheral tissue in insulin-mediated glucose uptake as well as glycogen and triglyceride storage [120, 123]. Therefore, skeletal muscle homeostasis is not only important in health but also in disease. Beyond the extensive research in central atherosclerosis, recent studies have focused on the effect of atherosclerosis on skeletal muscle, with most of the evidence emerging from human patients with peripheral arterial disease, as summarised in Table 2. PAD is a chronic degenerative condition that is characterised by vascular deficits leading to restriction and blockage of the arteries of the lower extremities and skeletal myopathy [5, 124]. The clinical manifestations of PAD are categorised in: i) intermittent claudication (IC), where leg pain occurs with physical activity but is alleviated at rest and ii) critical limb ischemia (CLI), where leg pain occurs at rest with or without tissue necrosis or gangrene [125, 126]. PAD is associated with increased cardiovascular morbidity and mortality and impaired quality of life. However, there is limited data (Table 3) on the underlying pathophysiology of skeletal muscle, which is often overlooked [127, 128]. Although restriction of blood flow was considered the most important factor of IC and CLI symptoms, recent studies suggest that it is probably not the only cause of functional impairment [124, 129–133]. Most emphasis has been given to the study of ischemia–reperfusion cycles that are considered an important contributor in skeletal muscle pathophysiology in PAD [134]. Emphasis is also placed on the pathophysiology of PAD and those factors that may lead to clinical manifestation of PAD including systemic inflammation, comorbidities, oxidative stress and mitochondrial deficits [125, 126, 128, 131, 135]. Current evidence on the effect of PAD in capillaries and skeletal muscle is discussed in this section (Fig. 1).

**Table 2:** Skeletal muscle deficits in patients with peripheral arterial disease

Reference	Intervention	Findings
[10]	62 ± 2 yr; IC > 1y; ABI: 0.58 ± 0.03; GT test → muscle biopsy of gastrocnemius	↓ maximal walking time, peak power, ABI, VO_2_ in PAD limbs; ↓ type I ↑ IIA fibres; ↔ IIB; 9% ↓ CSA; ↔ Capillary to fibre ratio; ↔ Muscle glycogen
[151]	68–71 yr; ABI: 0.64 ± 0.04; ↑ Smokers in PAD group; GT test → muscle biopsy of gastrocnemius	↓ Mitochondrial activity of complex I and III;
[27]	IC group with ABI = 0.35 ± 0.06; CLI group with ABI = 0.27 ± 0.05; Muscle biopsy of Gastrocnemius; More male in all groups; ↑ smokers, diabetes, hypertension in both PAD groups; ↓ obese in both PAD groups	Features of progressive muscle degeneration (i.e. myofibre atrophy, loss of the polygonal fibre shape, nuclear clumps, ↑ central nucleation, fibre vacuolization, target lesions, myofibre regeneration, myofibre necrosis and fibrosis and replacement of muscle by adipose tissue;
[190]	CLI; Study of popliteal arteries after amputation	↑ ET-1 plasma levels by 4-fold and ‘locally’ in femoral artery; ↑ atherosclerosis in popliteal arteries; ET-1 & receptors ET_A_ ET_B_ were associated with the luminal endothelium, ECs of the adventitial vasa vasorum and neural microvessels. ETA receptor similar in distribution to ET-1, co-localising with macrophages.
[148]	Diabetes, Hypercholesterolaemia, Hypertension; 76 yrs old; Grade III (intermediate), IV and V (advanced) lesions; Femoral artery samples	↑ inflammation in both stages, with different gene expression patterns; ↑ proteolysis and anti-proliferation markers & ↓ cell metabolism, catabolism in intermediate stage; ↑ vascular extracellular matrix markers & ↓ markers of protein folding, apoptosis, protein modification in advanced lesions
[150]	ABI = 0.55 ± 0.21; Age & sex matched; Diabetes, CAD, Dyslipidaemia, Hypertension; ↑ Smokers in PAD group; Muscle biopsy of gastrocnemius	↑ desmin by 21.5%; Abnormal morphology of myofibres with ↓ CSA, Negative correlation between CSA, morphology and desmin content; ↓ mitochondrial respiration from complex I and IV with irregular distribution; Negative correlation between isometric plantarflexor strength and desmin
[6]	Sex & Aged Matched; Type I Diabetes, CAD, Dyslipidaemia, Hypertension; ↑ Smokers in PAD groups Fontaine stage II (ABI = 0.53 ± 0.04) and IV (ABI = 0.25 ± 0.04); Muscle biopsy of gastrocnemius	↑ 25% of carbonyl content in all fibre types, mostly in type II fibres for all PAD patients; ↓ CSA; ↑ carbonyl content in PAD-IV vs. PAD-II patients; ↑ damage in type II fibres in PAD-II, whereas in PAD-IV type II and I/II had equal damage; Shift from type II to type I for both stages;
[141]	Age & Sex Matched; ↑ Smokers, Diabetes, CAD, Hypertension & Dyslipidaemia in PAD group; Advanced PAD ABI = 0.34 ± 0.05; Muscle biopsy of gastrocnemius	↓ activity of complexes I, III and IV; ↓ mitochondrial respiration; ↓ protein expression of MnSOD; ↑ Catalase, GPx activity; ↔ CuSOD activity; ↑ Carbonyl content, Lipid hydroperoxides and ﻿4-HNE adducts; ↔ Mitochondrial number;
[155]	Mean ABI = 0.4; Lower extremities operations	↔ mitochondrial respiration in baseline; ↓ mitochondrial respiration state 3 and 4
[9]	Age & sex matched; ↑ CAD & Hypertension in PAD groups; matched for smoking, myocardial infraction, stroke, renal insufficiency, obesity, dyslipidaemia; Fontaine Stage II, III and IV; ABI = 0.34 ± 0.24; Lower extremities operations	↑ carbonyl content and 4-HNE adducts in line with advanced stages of disease; ↓ myofibre CSA; ↑ oxidative damage
[192]	Age & Sex Matched; ABI = 0.6 ± 0.18; ↑ smokers and statin treatment in PAD group; Gardner Protocol; Muscle Biopsy of gastrocnemius	↑ apoptosis of endothelial cells in myofibres; ↑ caspase 3 expression;
[288]	Age matched; ABI < 0.9 for PAD patients; Smokers, non-diabetic; Muscle biopsy of gastrocnemius	↓ capillary density in gastrocnemius of PAD patients associated with ↓ VO_2max_, peak walking time and claudication onset time
[183]	Age & Sex Matched; Diabetic and smokers in both CLI and healthy control group; Muscle biopsy of gastrocnemius in CLI and control group followed by amputation in CLI group	↑ capillary density and capillary to myofibre ratio in gastrocnemius of CLI patients; abnormal structure of the capillaries in CLI group
[162]	ABI = 0.50 ± 0.17; Smokers, non-diabetic; Muscle biopsy of Gastrocnemius	↑ mtDNA deletion (4977 bp) deletion in both limbs but ↑↑ in the affected limb; ↑ other mtDNA deletions
[184]	Sex Matched; ↑ Age in PAD group; ABI = 0.64 ± 0.2; Muscle biopsy of gastrocnemius after exercise	↔ VEGFA concentration; ↔ VEGF_165_b; ↔ VEGFR1 concentration; ↓ capillaries in IC
[182]	Age matched; only male; CAD, Dyslipidaemia, Diabetes; Fontaine stage II-IV; ABI = 0.31 ± 0.25; Muscle biopsy of Gastrocnemius	↑ FoxO1 protein levels negatively correlated with ABI ↔ FoxO3a levels; ↑ p27^KIP1^ and THSB1
[147]	Aged matched; ↑ hypercholesterolaemia, smokers and male in PAD group; Fontaine stage III and IV; femoral and popliteal arteries	↑ serum PON1 and CCL2; ↑ thicker tunica intima/tunica media ratio in femoral arteries; ↑ calcium deposits in the media; ↑ PON1, PON3, CD68 (mainly in intima), CCL2 and its receptors DARC, CCBP2 not CCR2 in affected arteries
[142]	Fontaine stage IIa and IIb	↑ serum YKL-40 in PAD patients
[163]	Age & Gender Matched; ↑ hypertension in PAD group; ABI = 0.73 ± 0.14;	↓ capillary density and ↔ capillary/fibre ratio, CSA of fibres; ↑ thickening in basement membrane of lumina; ↓ volume of mitochondria
[143]	PAD with Fontaine stage III and IV	Correlation of PAD and serum cytokines for VEGF, CCL2 and TNFα
[144]	Only PAD patients with ABI = 0.72 ± 0.24 (no control group)	↑ gait impairment that correlated with hsCRP and ICAM-1
[191]	PAD-II (Fontaine stage II, ABI = 0.55 ± 0.22) and PAD-IV (stage IV, ABI = 0.22 ± 0.13) vs control group; Gender matched; Age matched only PAD-II vs control	↑ 3.5-fold in PAD-II and ↑ 8-fold in PAD-IV of TGFβ-1 vs Control; Correlation of collagen density and stage of PAD with TGFβ-1; TGFβ-1 was expressed only by sub-endothelial SMCs and associated with accumulation of fibroblasts and collagen deposition
[264]	PAD Fontaine Stage IIb vs control group;	↔ mRNA level and dialysate VEGF but ↓ protein in PAD vs control; ↔ mRNA levels of VEGFR-2, TSP1 and eNOS; ↑ dialysate of TSP1
[186]	PAD with/without CAD and control group; PAD patients with either IC (70%) or CLI (27%); Age/gender matched	↓↓ in PAD with/without CAD and ↓ in PAD with CAD of flow-mediated dilation and reactive hyperaemia
[132]	CLI; Amputated limbs	↑ calcification of media; lesions lacked of lipids and inflammatory cells, with atherosclerosis being present in less than 25%; Majority had type V, I and II lesions
[161]	Patients with IC and control group; ↑ dyslipidaemia, hypertension, CAD; age matched;	↓ glucose uptake from calf muscle in IC patients; glucose uptake correlates with whole body insulin resistance (with/without diabetes) and not ABI
[133]	PAD patients with IC and CLI (41%);	Majority had type V-VII plaques in femoral arteries; Type of lesions did not correlate with age, sex, diabetes and clinical stage; ↑ of inflammatory cells in lesions of CLI vs IC; No correlation between calcification and clinical stage; Correlation of SMCs and collagen deposition
[154]	PAD patients with IC (ABI = 0.63 ± 0.16) and control group; Age/gender matched; Muscle biopsy from Gastrocnemius	Heterogeneity in fibre type distribution in PAD; ↔ capillary density that doesn’t correlate with fibre type; ↓ SDH and COX-1 activity in myofibres due to ↑ autophagy of the intermyofibrillar mitochondria

Abbreviations and Symbols: *ABI* Ankle-Brachial Index, *CAD* Coronary Artery Disease, *CCBP2* Chemokine (C-C motif) binding protein 2, *CD68* Cluster of Differentiation 68, *CCL2* Chemokine (C-C motif) ligand 2, *CLI* Critical Limb Ischemia, *COX-1* Mitochondrial complex IV, subunit I, *CSA* Cross Sectional Area, *DARC* Duffy antigen/chemokine receptor, *EPC* Endothelial Progenitor Cells, *ET-1* Endothelin 1, *FoxO1* Forkhead Box Protein O1, *FoxO3* Forkhead Box Protein O3, *GT test* Graded treadmill test, *hCRP* high-sensitivity C-reactive protein, *IC* Intermittent Claudication, *ICAM-1* Intercellular adhesion molecule 1, *p27* ^*KIP1*^ Cyclin Dependent Kinase Inhibitor 1B, *PAD* Peripheral arterial disease, *PON1* Paraoxonase 1, *PON3* Paraoxonase 3, *SDH* Succinate dehydrogenase, *SMCs* Smooth Muscle Cells, *TGFβ-1* Transforming Growth Factor β 1, *THSB1/TSP1* Thrombospondin 1, *TNFα* Tumour Necrosis Factor α, *VCAM-1* Vascular Adhesion Molecule 1, *VEGF-A* Vascular Endothelial Growth Factor A, *VEGF* Vascular Endothelial Growth Factor, *VEGFR-2* Vascular Endothelial Growth Factor Receptor 2, *VO*_*2max*_ Maximal Oxygen Consumption, *YKL-40* Chitinase-3-like protein 1, ↑ increase, ↓ decrease, ↔ no change

**Table 3:** Overview of skeletal muscle deficits in intermittent claudication (IC) and critical limb ischemia (CLI)

Mild to Moderate Disease (IC)	Severe Disease - Critical Limb Ischemia
Oxidative stress mainly in type II fibres [6]	Oxidative stress in all fibre types [6, 27, 280]
↔/↓ capillary density, ↓ ABI (0.5–0.8) [154, 163, 288]	↓↓ ABI (<0.4) [141]
↓ mitochondrial volume [163]	Mitochondriopathy [141]
↑ TGFβ1, desmin accumulation [150, 191]	↑↑ TGFβ1, collagen [133, 191]
↔/↓ myofibre CSA [10, 163]	Fibrosis, Fat accumulation, different myofibre sizes but ↓↓ CSA [27]

Abbreviations and Symbols: *ABI* Ankle-Brachial Index, *CLI* Critical Limb Ischemia, *CSA* Cross Sectional Area, *IC* Intermittent Claudication, *TGFβ-1* Transforming Growth Factor beta 1; ↑ increase, ↓ decrease, ↔ no change

### Oxidative stress

As discussed above, central atherosclerosis is a chronic inflammatory disease that leads to plaque formation and occlusion of major arteries, initiated by increased oxidative stress and pro-inflammatory signalling that lead to endothelial dysfunction [2, 5, 136, 137]. However, accumulating evidence indicates that these two processes are inextricably intertwined and oxidative stress promotes cellular inflammation not only in aorta but also in skeletal muscle. This is partially attributed to a mechanism mediated by nuclear factor kappa-light-chain-enhancer of activated B cells (NF-κB) that promotes pro-inflammatory signalling and in turn amplifies oxidative stress via the production of reactive oxygen species [138]. Certain levels of ROS that are produced during muscle contraction are considered as important regulators of muscle function by activating pathways that regulate key homeostatic processes including myogenesis, muscle remodelling, regeneration, protein synthesis and substrate metabolism [139]. For example, ROS regulate glucose metabolism by activating the 5′adenosine monophosphate-activated protein kinase and initiating the mitogen-activated protein kinase signalling cascade [139]. However, excessive ROS production is deleterious and leads to oxidative damage in the muscle and may impact not only on cellular function but also damage DNA, lipids and proteins [138–140]. A fine tuning in ROS production exhibits opposing effects on muscle homeostasis and has important implications in health and disease states.

In fact, limb skeletal muscles of PAD patients exhibit increased protein carbonylation and 4-hydroxy-2-nonenal (4-HNE) adducts, which increase further as the disease advances [6, 9, 141]. It is worth mentioning that in the early stages of PAD (Fontaine stage II) type II myofibres seem to be more susceptible to oxidative damage than the hybrid type I/II and type I myofibres as judged by higher protein carbonyl contents [6]. Accordingly, in severe PAD (Fontaine stage IV) all myofibre types, i.e. type II, II/I and I, have similar levels of oxidative damage [6]. The oxidative damage of myofibres is evidenced by the elevated levels in carbonyl content, 4-HNE adducts and lipid hydroperoxides, despite an increase in protein expression of key antioxidants such as catalase and glutathione peroxidase, possibly due to a potential compensatory mechanism [141].

### Inflammation and apoptosis

Apart from increased oxidative damage, inflammation is another major initiator of central atherosclerosis. The majority of studies have focused on identifying markers of systemic inflammation and their potential role in PAD pathophysiology [135, 142–144]. Patients with PAD have elevated plasma levels of C-reactive protein and inflammatory markers such as monocyte chemotactic protein 1, D-Dimer (a fibrin degradation product), interleukin 6 (IL-6), TNFα, soluble VCAM-1 and soluble iCAM-1 are associated with an inferior performance in the 6 min walking test [5, 143–147]. To the best of our knowledge, studies investigating inflammation in the skeletal muscle of PAD patients remain to be conducted since the existing evidence is limited to the arteries of the affected limbs.

Inflammation in lesions of femoral arteries is increased in patients with CLI as compared with patients with IC, is mainly localised in the intima and is characterised by elevated number of macrophages [133, 147]. Immune system-related genes are upregulated in PAD and gene microarray analysis reveals different gene expression patterns in different stages of the disease [148]. There is also increased expression of genes regulating apoptosis (e.g. myristoylated alanine-rich C kinase substrate, *MARCKS* and phospholipid transfer protein, *PLP*), proteolysis (e.g. cathepsin B, *CTSB* and cathepsin C, *CTSC*) and immune function (e.g. Triggering receptor expressed on myeloid cells 1, *TREM1* and Gamma-interferon-inducible lysosomal thiol reductase, *IFI30*) in intermediate lesions, whereas genes controlling cell metabolism (e.g. glycine cleavage system protein H, *GCS* and phospholipase A2 group IVA, *PLA24A*) are downregulated [148]. In advanced lesions, vascular extracellular matrix gene expression is upregulated (e.g. Collagen Type I Alpha 1 Chain, *COL1A1* and Collagen Type III Alpha 1 Chain, *COL3A1*) and a downregulation of genes involved in cell cycle (e.g. cysteine and histidine rich domain containing 1, *CHORD1* and Neural precursor cell expressed developmentally down-regulated protein 9, *NDD9*) apoptosis (e.g. Metallophosphoesterase Domain Containing 2, *MPPED2* and Gutaminase, *GLS*) and RNA metabolism (e.g. Double-stranded RNA-specific adenosine deaminase, *ADAR*) is observed [148]. Another difference between the intermediate and advanced stages of PAD is that of the major histocompatibility complex class II molecules –involved in human leukocyte antigen mediated immune activation–are upregulated in intermediate lesions whereas in advanced lesions they are not differentially expressed [148].

### Mitochondrial deficits

Beyond oxidative damage and systemic inflammation, patients with PAD develop mitochondriopathy [9, 141]. Mitochondria are the major organelles for energy production and dysregulation of their function is associated with several diseases [149]. A variety of morphological changes ranging from irregular distribution, hypertrophy, transverse reorientation and lipid vacuolisation has been reported for the skeletal muscle mitochondria in PAD patients [7, 150]. As a result, several studies conclude that mitochondrial respiration in PAD muscles is compromised [7, 141, 151]. Electron transport chain complexes (ETC) I, III and IV activities have been shown to decrease either simultaneously or in various combinations [141, 150, 151]. Differences in the activity of ETC complexes may be due to the severity of PAD, since the average ancle-brachial index of the patients varied from 0.64 (intermediate stage) to 0.34 (severe stage) [141, 150, 151]. Similarly, in a mouse model of chronic ischemia, there is reduced enzymatic activity of complex III and decreased mitochondrial respiration [152, 153]. In a recent study, defective mitochondrial autophagy in type I fibres was reported in IC patients [154]. The authors suggested that deficiency in the intermyofibrillar mitochondrial activity is probably due to an increase in mitochondrial autophagy rather than a decrease in mitochondrial respiration [154]. Furthermore, in patients with severe PAD (with an average of ancle-brachial index 0.4) mitochondrial respiration remains the same at baseline prior to addition of substrates, whereas state 3 (i.e. ADP-stimulated respiration in the presence of excess substrate) and state 4 respiration (i.e. respiration in the absence of ADP and in presence of a single substrate) are decreased [155]. Despite the decrease in mitochondrial respiration, cytochrome c oxidase activity may be within the normal range [150]. The metabolic dysfunction of the skeletal muscle mitochondria in vivo has been shown with the accumulation of acylcarnitines not only in muscle but in plasma as well [7, 156]. Several studies have reported altered muscle metabolism in IC based upon decreased post-exercise recovery rate of phosphocreatine. This decrease is attributed to reduced oxygen and not to deficient oxidative phosphorylation [157–160]. Apart from changes in oxidative metabolism, PAD patients with whole body insulin resistance exhibit decreased glucose uptake in the calf muscle [161]. Due to increased oxidative stress in the muscle, mitochondrial damage has also been reported in PAD muscle. The mitochondrial genome is prone to oxidative injury as well as an accumulation of mitochondrial DNA deletions, which have been reported in PAD patients, with the 4977 bp deletion being the most common [162]. Thus, it appears that mitochondrial damage commences at an early stage of the disease and aggravates with disease progression and it may correlate to increased oxidative stress.

### Altered myofibre morphology and fibrosis

The myopathy in PAD is also characterised by the alteration in the myofibre morphology. Several groups have reported that myofibres reduce in size as the disease advances and the loss in cross sectional area (CSA) is correlated with the increase in oxidative damage [9, 11, 150, 151]. However, others have failed to report decreases in CSA, possibly due to differences in disease severity among studies [6, 27, 163]. Along with the decrease in size, myofibres lose their polygonal shape [6, 9]. As PAD advances from IC to CLI, a decrease in muscle force generating capacity is observed and muscle degeneration proceeds with the appearance of nuclear clumps, central nucleation, myofibre regeneration/degeneration, a substantially wider range of sizes of the myofibres, fibrosis and fat deposition [27]. Similar changes such as reduced CSA, loss of the polygonal shape, wide range of myofibre size and central nucleation are features of the myopathy in the ischemic mouse model with ligation of both femoral and iliac arteries [152, 153]. Skeletal muscle satellite cells are a local stem cell population that are indispensable of skeletal muscle fibre regeneration and repair after injury [164, 165]. Satellite cell number decreases with age and their regenerative capacity has been shown to either reduce or remain unaltered [166–168]. The role of satellite cells in skeletal muscle regeneration of PAD has not received full attention so far and remains to be established in human studies [169]. However, it can be assumed that the regenerative capacity of satellite cells may be limited due to the decline of satellite cell number with ageing along with the PAD pathophysiology i.e., ischemia in combination with systemic inflammation, denervation, mitochondrial dysfunction and increased oxidative stress and comorbidities (i.e. diabetes, hyperlipidaemia) [168–174]. A few studies in preclinical models of PAD with hindlimb ischemia have attempted to elucidate the regenerative capacity of satellite cells [175–180]. On one hand, prolonged hypoxia was shown to delay skeletal muscle regeneration, due to impairment of satellite cell differentiation [177, 178]. On the other hand, strategies that attenuate oxidative stress seem to augment the regenerative capacity of satellite cells [179, 180]. Notably, most of these studies have been conducted in young and healthy mice without any comorbidities, where the satellite cell potential is influenced only by ischemia. Overall, the majority of studies on the regenerative potential of satellite cells in PAD is based on preclinical experimental models and extrapolation of findings to humans should be made with caution [181].

### Capillary density, chronic ischemia and impaired oxygen supply

Atherosclerosis in the arteries of the lower limbs leads to chronic ischemia of the muscle due to reduced oxygen delivery [5]. Nonetheless, capillary density in PAD patients with intermittent claudication has been reported to remain unaltered or reduced [5, 163, 182]. In patients with critical limb ischemia (CLI), capillary density is further reduced, although some groups report an increase of microvessel density when compared to intermittent claudication, however the increase might be attributed to the loss of myofibres [5]. Moreover, the microvessels in CLI have abnormal structure and lose their proliferative capacity [183]. VEGFA and the angiostatic VEGF165b factor seem to remain unchanged in intermittent claudication whereas VEGF receptor 1 is decreased [184]. However, an increase has been shown in VEGFA and VEGF receptor 2 in the atrophic myofibres which is local and correlates with the presence of inflammatory cells [185]. Angiostatic factors that are elevated in the muscle of patients with severe PAD are forkhead box protein O1 and matrix protein thrombospondin 1, whereas mRNA levels of VEGF, VEGF receptor 2, angiopoietin receptor 2 and HIF1α have a small increase [5, 182].

PAD patients exhibit vascular endothelial dysfunction not only in the large vessels but also in the microvasculature [186–188]. ET-1 is a potent vasoconstrictor that is produced by endothelial cells and has been shown to contribute to the atherosclerotic narrowing of the lower extremities [189]. Plasma levels and signalling of ET-1 are both elevated in CLI patients by 4-fold; ET-1 expression is increased in femoral artery and the endothelin 1 receptor A is co-localised with macrophages in the plaque region [190]. In patients with IC, the basement membrane of the lumina is thicker and may play a role in impaired exercise tolerance [163]. Vascular smooth muscle cells in IC express high levels of transforming growth factor beta 1 (TGFβ1), a pro-fibrotic cytokine [191]. As PAD progresses, the tunica media of affected femoral arteries becomes thicker, there is collagen deposition and fibroblast accumulation in the microvasculature [163, 191]. Collagen deposition and TGFβ1 levels correlate with the PAD stage [133, 191]. Apart from endothelial dysfunction and collagen deposition in patients with intermittent claudication, endothelial cells in the microvasculature have increased expression of caspase 3 and elevated levels of apoptosis [192].

Consequently, peripheral atherosclerosis impacts on skeletal muscle as outlined by increased oxidative damage, inflammation, mitochondriopathy, loss of number and function of myofibres, impaired capillary density and apoptosis. Although ischemia– reperfusion cycles are considered the principal cause of skeletal muscle pathophysiology in PAD, the impact of other comorbidities such as hyperlipidaemia on endothelial dysfunction and skeletal muscle pathophysiology should not be overlooked.

## Experimental evidence on the skeletal muscle pathophysiology of atherosclerotic mice

Although ApoE^-/-^ and LDL receptor deficient (Ldlr^-/-^) mice are widely used as models for systemic atherosclerosis, currently there is sparse scientific evidence evaluating the skeletal muscle pathophysiology of these two experimental models of atherosclerosis, as summarised in Table 4 [46, 193]. ApoE^-/-^ mice on normal chow diet have decreased capillary density in the gastrocnemius from the age of 12 weeks and nitric oxide bioavailability in skeletal muscle arterioles declines from the age of 20 weeks without changes in endothelial function [194, 195]. At 65 weeks of age ApoE^-/-^ mice exhibit extensive plaque formation in the aorta and atherosclerotic plaques are also found in the femoral arteries [196]. Blood perfusion does not seem to be compromised between 2.5 and 8 months of age, while exercise capacity either remains unaltered or decreases [196, 197]. Curiously, one study reported a beneficial phenotype with regard to inflammation, insulin sensitivity and intramuscular lipid contents in ApoE^-/-^ mice administered a high-fat diet (i.e. 21% w/w fat corresponding to 40% kcal from fat) compared to wild type mice [198]. This striking finding was attributed to a possibly attenuated triglyceride-rich lipoprotein-derived fat delivery to skeletal muscle due to ApoE deficiency, but mechanistic insights remain to be established and validated in future studies. On the other hand, ApoE^-/-^ mice on a different high-fat diet consisting of a higher percentage of fat (60% v/v fat) exhibit elevated inflammation and greater levels of hydrogen peroxide in the muscle as compared with wild type mice [199]. Ldlr^-/-^ mice at the age of 22 weeks have decreased capillary density in the gastrocnemius, which is less pronounced than in the ApoE^-/-^ and the wall to lumen ratio is elevated in the skeletal muscle arterioles [194]. Taken together these data suggest that although there may be decreased capillarisation in the muscle of the murine atherosclerotic models, neither glucose uptake nor inflammation seems to be altered, despite the limited evidence so far. The absence of characterisation of skeletal muscle morphology and other biological parameters such as oxidative stress and mitochondrial function remain to be elucidated before valid conclusions can be drawn.

**Table 4:** Experimental evidence with manifestations of atherosclerosis in skeletal muscle

Reference	Intervention	Findings
[195]	♂ ApoE^-/-^, Ldlr^-/-^ and WT (20wks); ND	↑ arterial pressure& Insulin resistance in Ldlr^-/-^; ↔ endothelial vasodilation and VSMC reactivity in skeletal muscle arterioles; ↓ NO bioavailability in ApoE^-/-^
[194]	♂ ApoE^-/-^, Ldlr^-/-^ and WT (12–13 and 22–23 wks); ND	↓ capillary density in gastrocnemius of ApoE^--/-^ from 12wks and in Ldlr^-/-^ from 22wks; ↑ plasma oxidative stress and inflammatory markers in ApoE^-/-^ and Ldlr^-/-^; ↑ wall:lumen ratio in Ldlr^-/-^
[198]	♂ ApoE^-/-^ and WT (18wks); HFD (21% w/w) or ND for 12wks	↓ inflammation in AT and skeletal muscle of ApoE^-/-^ HFD; ↓ Akt phosphorylation in AT and skeletal muscle of WT HFD; ↑ crown like structures in WT HFD; ↓ dietary lipid incorporation in adipose tissue, skeletal muscle and liver in ApoE^-/-^ HFD; No oxidative stress in adipose tissue; ↑ TGs in skeletal muscle of WT HFD
[199]	♂ ApoE^-/-^ and WT (16wks); HFD (60% v/v fat) or ND for 9wks	↑ H_2_O_2_ in liver and muscle of ApoE^-/-^ ND and HFD; ↑ H_2_O_2_ in AT of ApoE^-/-^ HFD; ↑ IL-6 in adipose tissue of WT HFD and ApoE^-/-^; ↑ TNFα of WT HFD and ApoE^-/-^ HFD; ↑ crown like structures in adipocytes of ApoE^-/-^ HFD
[215]	♀ ApoE^-/-^ (8-10months); Femoral artery ligation; ND	Hyperaemic response to treadmill exercise similar to human studies on PAD
[211]	ApoE^-/-^ (19-21wks); Iliac artery ligation; ND	↓ blood flow in the ischemic hindlimbs; ↑ capillarisation only in quadriceps; ↑ fibre atrophy; ↑ of glucose uptake and pro-inflammatory macrophages and T cells at early ischemic stages
[217]	♀ ApoE^-/-^ and WT (8-10months); Hindlimb ischemia; ND	↓ myogenin levels in 7d post and ↑ MCP-1 levels at 14d post ischemia/reperfusion in ApoE^-/^; Delayed skeletal muscle regeneration
[218]	♂ and ♀ ApoE^-/-^ and WT (18-26wks); Notexin injury; ND	↑ fat lipid deposition and calcification; ↓ fibre size; Delayed skeletal muscle regeneration
[216]	♀ ApoE^-/-^ and WT (16-8wks); ND for WT and WD (1.25% cholesterol, 15% w/w fat) in ApoE^-/-^ for 10wks; Hindlimb ischemia (removal of femoral artery and all major collateral branches) and subsequent treatment for 21d with miR-150 mimic peptide	↓ blood perfusion in ApoE^-/-^ vs WT that improved with the miR-150 peptide; ↑ ambulatory impairment in ApoE^-/-^ vs WT that decreased with the miR-150 peptide; ↓ capillary density in ApoE^-/-^ vs WT that improved with the miR-150 peptide; ↓ number and functional activities of PACs in ApoE^-/-^ vs WT that improved with the miR-150 peptide
[221]	♀ Ldlr^-/-^ (22wks) and ApoE^-/-^ (36wks); Hindlimb ischemia (double ligation of femoral artery) and subsequent treatment with IFNAR1 Mab or IgG isotype (control); WD (16% w/w fat, 0.15% cholesterol) in Ldlr^-/-^ for 8wks and for 24wks in ApoE^-/-^	↑ hindlimb perfusion restoration in Ldlr^-/-^ treated with IFNAR1 Mab vs Ldlr^-/-^; ↔ capillary density in Ldlr^-/-^ treated with IFNAR1 Mab vs Ldlr^-/-^; ↔ atherosclerotic burden and lesion characteristics in Ldlr^-/-^ treated with IFNAR1 Mab vs Ldlr^-/-^; ↑ hindlimb perfusion restoration in ApoE^-/-^treated with IFNAR1 Mab vs ApoE^-/-^; ↓ number of arterioles in ligated limb of ApoE^-/-^ treated with IFNAR1 Mab and ApoE^-/-^ vs sham side of ApoE^-/-^ treated with IFNAR1 Mab and ApoE^-/-^ respectively; ↔ atherosclerotic burden and lesion characteristics in ApoE^-/-^ treated with IFNAR1 Mab vs ApoE^-/-^
[220]	♂ Ldlr^-/-^, Ldlr^-/-^/CCR7^-/-^, WT (8-12wks); Hindlimb ischemia with electrocoagulation of femoral artery	Blood flow recovery at 7d for WT, at 10d for LDLR^-/-^ and in Ldlr^-/-^/CCR7^-/-^ was not fully recovered after 21d; ↔ number of dendritic cells and T lymphocytes in spleen, lymph nodes and blood in Ldlr^-/-^ vs WT
[219]	♂ Ldlr^-/-^ and WT (18wks); WT on ND and Ldlr^-/-^ on HFD (15.1% fat, 1.25% cholesterol) for 12wks; Iliac and femoral artery ligation at 18wks; Subsequent treatment with heparin, bFGF, heparin and bFGF or no treatment for 4wks	Blood flow of ischemic limb: ↑ in heparin treated, ↑↑ in bFGF treated and ↑↑ in bFGF plus heparin in WT vs WT not treated and in Ldlr^-/-^: ↔ in heparin, in bFGF and not treated vs ↑ in bFGF plus heparin; Blood perfusion in ischemic limb: ↓ in not treated Ldlr^-/-^ vs WT not treated; Mature vessels of ischemic limb: ↑ in heparin treated, ↑ in bFGF treated, ↑ in bFGF plus heparin in WT vs WT not treated and Ldlr^-/-^ : ↑ in heparin, ↑ in bFGF and ↑↑ bFGF plus heparin vs Ldlr^-/-^not treated

Abbreviations and Symbols: *Akt* Protein kinase B, *ApoE* ^*-/-*^ Apolipoprotein E knockout, *AT* Adipose Tissue, *bFGF* basic Fibroblast Growth Factor, *CCR7* Chemokine C-C receptor type 7, *HFD* High-fat Diet, *H*_*2*_ *O*_*2*_ Hydrogen Peroxide, *IFNAR1* Interferon α/β receptor type 1, *IL-6* Interleukin 6, *Ldlr* ^*-/-*^ Low-Density Lipoprotein Receptor knockout, *Mab* Monoclonal Antibody, *MCP1* monocyte chemotactic protein 1, *ND* Normal Diet (chow diet), *NO* Nitric Oxide, *PACs* Bone marrow derived proangiogenic cells, *PAD* Peripheral Arterial Disease, *TBARS* Thiobarbituric acid reactive substances, *TNFa* Tumor necrosis factor a, *VSMC* vascular smooth muscle cells, *WD* Western-type diet, *WT* wild type; ↔ similar, ↓ decrease, ↑ increase; ♂: male; ♀: female; All strains WT, ApoE^-/-^ and LDLR^-/-^ are on a C57Bl/6 background

The study of skeletal muscle pathophysiology in PAD has been modelled in animal studies using the “femoral artery ligation” model of hind-limb ischemia, with the majority of data being obtained from young and healthy animals in the absence of any cardiovascular risk factors or comorbidities associated with PAD such as hyperlipidaemia [152, 196]. These studies have provided valuable information on the role of inflammatory and angiogenic factors in skeletal muscle regeneration [200–204]. However, it has to be taken into consideration that revascularisation (angiogenesis and arteriogenesis) following femoral artery ligation in mice is rather a rapid process, since blood perfusion is restored within a month after an ischemic injury and cannot adequately simulate chronic human PAD [152, 196]. Interestingly, when femoral ligation is combined with iliac artery ligation, it has been shown to simulate a more stable PAD model over-time with chronic ischemia resembling severe human PAD [152]. This model shows increased ROS production, protein carbonyl content and 4-HNE adducts that indicate oxidative stress, as well as decreased enzymatic activity of the mitochondrial antioxidant superoxide dismutase 2 (SOD2) and mRNA levels of the antioxidants catalase, superoxide dismutase 1 and 2, despite increased SOD2 protein expression levels [152, 153]. It is also worth mentioning that there is no available model to fully recapitulate the pathophysiology of human PAD, not only due to the existence of comorbidities in PAD patients (such as hyperlipidaemia, diabetes and hypertension), but also due to the intra-individual heterogeneity of the clinical disease [205]. The reader is directed to two interesting recent reviews that discuss the challenges of translating experimental evidence into human disease [126, 205]. In brief, in the femoral or iliac artery ligation model the response to ischemia is not similar to the human chronic PAD with animal models exhibiting, as mentioned earlier, an intense collateralisation that contributes to a rapid restoration of blood flow and recovery of muscle function which is not evident in PAD [201, 205]. This has been attributed to the formation of pressure gradient between the ischemic and non-ischemic limb that causes an increase in shear stress and blood flow in the collateral arteries. In combination with the hypoxic environment, increased blood flow promotes infiltration of inflammatory cells that induce angiogenesis, arteriogenesis and myogenesis by enhancing expression of VEGF upstream regulators, such as HIF-1α and NF-κB [127, 206–208]. Moreover, muscle tissue necrosis in animal models occurs in a greater degree than in PAD patients, where chronic inflammation accompanied by insufficient collateralisation lead to replacement of the damaged tissue with fibrotic tissue [201, 209]. The aforementioned differences between acute arterial occlusion and PAD disease may at least in part account for the unsatisfactory results of angiogenic therapies in clinical trials [205, 210]. On the other hand, gradual arterial occlusion with use of ameroid constrictors is considered a potentially better model for mimicking human ischemic disease, since it does not induce tissue necrosis [209]. In addition, blood flow recovery and inflammation are less profound compared to the acute arterial occlusion model [209]. Apart from the type of ischemia, the presence of comorbidities is also an important difference. Most PAD models comprise of healthy young models, whereas development of PAD in human patients is secondary to atherosclerosis and coexists with several cardiovascular factors [211]. Since mouse models of PAD are based on artery ligation for induction of ischemia, these models cannot entirely mimic human pathology in terms of fibrothrombotic lesion formation given that acute arterial embolism does not occur spontaneously [212]. Experimental femoral artery ligation mouse models with either hypercholesterolaemia, metabolic syndrome, hypertension or type 1 diabetes have delayed blood perfusion when compared to wild type mice [213]. Thus, future studies should take into account that a chronic model of ischemia with comorbidities would be more pertinent than the acute ischemia model [126, 202].

In this regard, a few studies have used ApoE^-/-^ mice as a model for PAD by inducing limb ischemia via femoral artery ligation [211, 214, 215]. Specifically, ApoE^-/-^ mice subjected to ischemia followed by exercise have similar hyperaemic response with exercising PAD patients and exhibit delayed regeneration and increased inflammation [214, 215]. In a recent study ApoE^-/-^ mice on a western-type diet and subsequent induced-ischemia, display impaired ambulation compared to wild type mice due to delayed restoration of blood perfusion [216]. In the hindlimb ischemia-reperfusion injury model, ApoE^-/-^ mice exhibit impaired muscle regeneration [217]. Similarly, in notexin-induced muscle injury ApoE^-/-^ mice display impaired myofibre regeneration with increased fat infiltration and calcification. The authors concluded that the delay in muscle healing is due to impaired macrophage phagocytic activity [218]. Ldlr^-/-^ mice are also used as a model for PAD by ligating the femoral artery and inducing limb ischemia [219–221]. Ldlr^-/-^ mice on a western-type diet and subjected to ligation of both femoral and iliac arteries have reduced blood perfusion as compared to wild type mice [219]. In a different model of ischemia induced by coagulation of the femoral artery, Ldlr^-/-^ mice show complete blood flow recovery at 10 days after injury, whereas wild type mice recover fully after 7 days [220]. However, a direct comparison of genotypes in this study was not performed. Taken together these data suggest that both experimental models of atherosclerosis are suitable for the study of PAD. To the best of our knowledge no studies have compared both atherosclerotic mouse models of the same age and diet to draw certain conclusions as to which model may be more beneficial for the study of PAD.

## NADPH Oxidases in vascular disease

Reactive oxygen species are a diverse class of reactive chemical molecules that consist of highly reactive oxygen atoms [222]. ROS play an important role in multiple physiological processes by regulating enzymatic activities, transcription factors and nucleic acids that are involved in cell growth, proliferation and survival [222]. ROS are usually produced as a ‘by-product’ by diverse sources in the cell, such as respiratory enzymes, lipooxygenases, eNOS and xanthine oxidase [25, 222]. NADPH oxidases are the only enzymes that produce primarily ROS [25]. There are 7 homologues of Noxs that have been identified in the mammalian species, specified Nox1 to Nox5 and Dual Oxidases (Duox) 1 and 2 [223]. Noxs are multimeric protein complexes and the majority of them produces superoxide via their catalytic Nox subunit that is membrane bound and transfers electrons from cytosolic NADPH to molecular oxygen [25, 222]. Nox4, Duox1 and 2 seem to produce hydrogen peroxide due to dismutation of superoxide [222]. Nox1 to Nox3 enzymatic activities are regulated by the cytosolic proteins NADPH oxidase activator 1 (Noxa1) and NADPH oxidase activator 2 (p67phox) as well as scaffolding proteins that attach the cytosolic activators to the Nox catalytic subunits neutrophil cytosol factor 1 (p47phox), neutrophil cytosol factor 4 (p40phox) and NADPH oxidase organiser 1 (Noxo1) [224]. Nox1 interacts preferentially with Noxa1 (p67phox homologue) and Noxo1 (p47phox homologue) but can interact with p47phox and Nox2 interacts mainly with p47phox and p67phox [224]. The GTPases Ras-related C3 botulinum toxin substrate 1 and 2 (Rac1 and Rac2) are important for Nox activation, apart from Nox4 that is constitutively active [223]. However, Noxs as monomers are inactive and rely on the interaction with the transmembrane scaffolding protein superoxide-generating NADPH oxidase light chain subunit (p22phox) to be activated, with the exception of Nox5, Duox 1 and 2 [223, 224].

Noxs were first identified in phagocytic cells and they are responsible for the respiratory burst that is essential for innate immune response and phagocytosis of pathogens [25, 222]. In the vascular wall, Noxs are a significant source of ROS production. Endothelial cells express Nox1, Nox2, Nox4 and Nox5, vascular smooth cells express Nox1, Nox4 and Nox5 while adventitial fibroblasts express Nox2 and Nox4 [25, 222]. In vascular diseases such as atherosclerosis, in diabetes and hypertension, there is an increase in ROS production from the vasculature and is considered an important initiator of atherogenesis by promoting proinflammatory pathways and oxidative stress that lead to endothelial dysfunction [25, 225, 226]. Indeed, with the use of transgenic and knockout mice Nox1 and 2 were shown to play an important role in atherosclerosis [57, 226]. Consequently, the role of Noxs in atherogenesis has lately received much attention as summarised in Table 5 [226, 227]. ApoE^-/-^/p47phox^-/-^ double knockout mice at the age of 16 weeks have similar total plasma cholesterol levels and decreased vascular superoxide production but the same lesion area in the aortic sinus with ApoE^-/-^ mice [228]. Although the lesion area is similar in the aortic sinus, ApoE^-/-^/p47phox^-/-^ double knockout mice at the age of 30 weeks have reduced lesion area by 75% in the whole aorta and under a high-fat diet the reduction reaches 50% at the age of 18 weeks [90]. Allogenic bone marrow transplantation between ApoE^-/-^/p47phox^-/-^ and ApoE^-/-^ followed by a high-fat diet (42% kcal from fat) leads to decrease of lesion coverage (>50%), smaller lesion size, fewer macrophages and lower endothelial superoxide production in both groups [229]. ApoE^-/-^ mice with bone marrow from ApoE^-/-^/p47phox^-/-^ have lower levels of oxidised LDL and ApoE^-/-^/p47phox^-/-^ with bone marrow from ApoE^-/-^ have reduced expression of cellular adhesion molecules. These findings suggest that on one hand the NADPH oxidase activity contributes to lesion formation and on the other hand vascular wall cells and bone marrow promote atherogenesis through different processes [226, 229]. Although the studies on p47phox deficiency give an insight into the role of Noxs in atherosclerosis, it is difficult to dissect the particular role of individual Nox homologues to the above findings, since Nox2 interacts mainly with p47phox and Nox1 mainly with Noxo1 but the interaction of Nox1 with p47phox cannot be ruled out [226].

**Table 5:** Targeting NADPH oxidases in skeletal muscle

Reference	Intervention	Findings
[94]	♂ ApoE^-/-^ (16wks); ND or HFD (15.8% fat, 1.25% cholesterol) for 8wks; For 4wks; HFD + Nox2 inhibitor peptide (Nox2ds-tat) or HFD plus control sequence (scrambled, Scr)	↑ cholesterol and TGs in Scr and Nox2ds-tat; ↑ O_2_^.-^ production in carotid arteries and atherosclerotic lesions throughout the aorta of HFD and Scr but ↓ in Nox2ds-tat; ↓ mRNA expression of p47phox and p22phox in Nox2ds-tat vs Scr; ↑ gene expression of VEGF, HIF1α, visfatin and MMP9 in carotid arteries of Scr, ↓ in Nox2ds-tat; ↑ MMP9 activity and protein levels in carotid arteries of Scr vs ND and ↓ in Nox2ds-tat
[90]	♂, gp91^-/-^, ApoE^-/-^ and ApoE^-/-^/p47^-/-^ (18wks); ND and HFD for 10wks (15% w/w fat, 34% kcal from fat)	↓ p47^-/-^ in gp91^-/-^ response of SMC in growth factors in vs WT; ↓ O_2_^.-^ expression in aortas of p47^-/-^ vs WT; ↔ ApoE^-/-^ and ApoE^-/-^/p47^-/-^ in serum lipid levels; ↓ lesion area throughout the aorta, ↔ in the aortic sinus in ApoE^-/-^/p47^-/-^ vs ApoE^-/-^ HFD
[228]	♂ ApoE^-/-^, ApoE^-/-^/p47^-/-^ (16wks); ND	↔ basal O_2_^.-^ levels in aorta that ↓ in ApoE^-/-^/p47^-/-^ after inhibition of SOD; ↔ aortic lesion area, serum blood levels and aortic blood pressure
[233]	♂ and ♀ WT, gp91^-/-^ (30wks), ApoE^-/-^, ApoE^-/-^/gp91^-/-^ (24wks); HFD for 20wks in WT and gp91^-/-^ (15% w/w, 37.1% kcal from fat, 1.25% cholesterol, and 0.5% sodium cholate); ApoE^-/-^and ApoE^-/-^/gp91^-/-^ in ND	↓ O_2_^.-^ production from peritoneal macrophages in gp91^-/-^ and WT; ↔ gp91^-/-^ and WT in plasma lipid profile and lesion area (♀: ↓ plasma TGs and ↑ lesions); ↓ plasma cholesterol & TGs (only in ♂ ApoE^-/-^/gp91^-/-^ vs ApoE^-/-^); ↓ 2-fold HDL and ↑ 60% of LDL in ApoE^-/-^/gp91^-/-^ vs ApoE^-/-^; ↔ aortic sinus lesion area in ApoE^-/-^/gp91^-/-^ vs ApoE^-/-^
[57]	♂ApoE^-/-^ & (12 and 19wks); WD (21% w/w fat, 40% kcal and 0.15% cholesterol) for 7 and 14wks	↓ superoxide production, ↑ NO bioavailability and ↓ lesion coverage in Nox2^-/y^ApoE^-/-^
[229]	♂ and ♀ ApoE^-/-^, ApoE^-/-^/p47^-/-^ ; Bone marrow transplanted mice for 4wks in ND and then in WD (42% kcal from fat, 0.2% cholesterol) for 12wks; (Control: Bone marrow transplantation from ApoE^-/-^ to ApoE^-/-^)	↓ VWO & BMO in atherosclerotic coverage, lesion size with fewer macrophages and O_2_^.-^production vs Control; ↔ BMO & VWO in total cholesterol, TGs, expression of p22phox and catalytic subunits of Nox1 and Nox4; ↓ BMO in oxLDL levels vs VWO and Control; ↓ VWO in gene expression and immunostaining of VCAM1, iCAM1 and P Selectin vs BMO and control ↓ ApoE^-/-^/p47phox^-/-^ in neointimal hyperplasia after femoral injury vs ApoE^-/-^; ↑ VSMC proliferation in ApoE^-/-^ vs ApoE^-/-^/p47phox^-/-^
[225]	ApoE^-/-^, Nox2Tg ApoE^-/-^(9-24wks); Sex, NS; ND	↑ O_2_^.-^ in Nox2Tg ApoE^-/-^; ↑ VCAM1 and macrophage recruitment in Nox2Tg ApoE^-/-^ only at 9wks; ↔ total plasma cholesterol, LDL, HDL, TGs, OxLDL lesion area, lesion progression and composition, macrophage recruitment and lipid deposition in Nox2Tg ApoE^-/-^; Treatment with AngII for 4wks ↑ lesions in a dose-dependent manner similarly
[231]	♂ ApoE^-/-^ and Nox1^-/y^ApoE^-/-^ (12, 19 and 26wks); WD (21% w/w fat and 0.15% cholesterol) for 7,14, or 21wks	↑ VLDL/LDL, TGs, O_2_^.-^ and intimal thickening in aortic sinus in Nox1^-/y^ApoE^-/-^; ↔ plaque area in Nox1^-/y^ApoE^-/-^ ; ↓ collagen, SMCs and ↑ MMP-9 in aortic sinus lesions in Nox1^-/y^ApoE^-/-^
[230]	♂ ApoE^-/-^ and Nox1^-/y^ApoE^-/-^ (24wks); HFD for 18wks (42% kcal from fat)	↓ aortic lesions, O_2_^.-^ production and number of macrophages in lesions in Nox1^-/y^ApoE^-/-^
[91]	♂ WT (28 wks old); HFD for 8wks (60% kcal from fat) or ND, a HFD subgroup treated with apocynin	↑ plasma insulin, glucose and HOMA-IR in HFD; ↓ glucose uptake from muscle and ↑ H_2_O_2_ in myofibres after insulin stimulation of HFD; GSH/GSSG ratio in muscle of HFD; ↑ Nox2 and p47phox protein expression in HFD muscle; ↓ insulin resistance in whole body and muscle and p47phox and Nox2 levels in muscle of HFD after treatment with apocynin
[239]	♂ WT and Nox2^-/y^ (18 and 42wks); ND or HFD (45% kcal from fat) for 3 or 9 months	↑↑ WT HFD and ↑ Nox2^-/y^ in BW, HOMA-IR and GTT; ↔ oxidative and glycolytic myofibres; ↑ Nox2, p22phox, p67phox and O_2_^.-^production in the muscle of WT HFD; ↑ Nox2^-/y^ HFD and ↓ WT HFD in Glut4 and Akt phosphorylation of muscle; ↑ Nox2, p22phox, p67phox and ↓ phosphorylated Akt and glucose uptake in myoblasts treated with palmitate or high concentration of glucose; ↓ phosphorylated Akt and glucose uptake in shRNA Nox2-expressing myoblasts after treatment with H_2_O_2_

Abbreviations and Symbols: *Akt* Protein Kinase B, *AngII* Angiotensin II, *ApoE* ^*-/-*^ Apolipoprotein E knockout, *ApoE* ^*-/-*^ */gp91* ^*-/-*^ Double Apolipoprotein E knockout and gp91 knockout, *ApoE* ^*-/-*^ */Nox1* ^*-/y*^ Double Apolipoprotein E knockout and Nox1 knockout, *ApoE* ^*-/-*^ */p47* ^*-/-*^ Double Apolipoprotein E knockout and p47 knockout, *BMO* Bone Marrow transplantation from ApoE^-/-^/p47phox^-/-^ to ApoE^-/-^, *BW* Body Weight, *DHE* Dihydroethidium, *Glut4* Glucose Transporter Type 4, *gp91* ^*-/-*^ gp91 knockout, *GSH* Glutathione reduced, *GSSG* glutathione oxidised, *GTT* Glucose Tolerance Test, *H*_*2*_ *O*_*2*_ Hydrogen Peroxide, *HDL* High-density lipoprotein, *HIF1a* Hypoxia Inducible Factor 1a, *HFD* High-fat Diet, *HOMA-IR* Homeostatic Model Assessment for Insulin Resistance, *iCAM1* Intercellular adhesion molecule 1, *LDL* Low-density lipoprotein, *MMP9* Matrix metalloproteinase 9, *Nox2* nicotinamide adenine dinucleotide phosphate-oxidase 2, *Nox2* ^*-/y*^ Nox2 Knockout, *Nox2Tg ApoE* ^*-/-*^ ApoE^-/-^ with overexpression of Nox2 specifically in Endothelial Cells, *ND* Normal Diet (chow diet), *NS* Not Specified, *O*_*2*_^*.-*^ Superoxide, *oxLDL* Oxidised LDL, *p47phox* ^-/-^ p47phox knockout, *shRNA Nox2-expressing* Short Hairpin RNA to silence Nox2 expression, *SOD* Superoxide Dismutase, *SMC* Smooth Muscle Cells, *TGs* Triglycerides, *VCAM1* Vascular cell adhesion molecule 1, *VEGF* Vascular Endothelial Growth Factor, *VLDL* Very low-density lipoproteins, *VSMC* vascular smooth muscle cells, *VWO* Bone Marrow transplantation from ApoE^-/-^ to ApoE^-/-^/p47phox^-/-^, *WD* Western-type diet, *WT* wild type; ↓ decrease, ↑ increase, ↔ similar; All strains WT, ApoE^-/-^, ApoE^-/-^/gp91^-/-^, gp91^-/-^, Nox2Tg ApoE^-/-^, Nox1^-/y^ApoE^-/-^, Nox2^-/y^ApoE^-/-^ and p47^-/-^are on a C57Bl/6 background

The role of Nox1 in atherosclerosis is not fully elucidated and there are contradictory data from studies on Nox1 depletion. Sheehan et al. reported that Nox1 deficiency in ApoE^-/-^ mice (Nox1^-/y^/ApoE^-/-^) fed a high-fat diet (42% kcal from fat) exhibited reduced superoxide production and a decreased lesion area by 35% in the aortic arch and 28% in the entire aorta. Furthermore, the lesions contained less macrophages [230]. On the contrary, Nox1^-/y^/ApoE^-/-^ mice fed a western-type diet exhibited similar lesion coverage throughout the aorta and the thickening of the intima was larger at the level of the aortic sinus compared to ApoE^-/-^ mice [231]. Additionally, the lesions in the aortic sinus appear to be more unstable implying that Nox1 has a protective role in atherosclerosis [231]. Similarly, Nox1 deficiency in the aortas of diabetic ApoE^-/-^ mice reduced lesion coverage, attenuated macrophage infiltration, expression of pro-inflammatory chemokines and ROS production, while it did not have any effect in ApoE^-/-^ mice [232]. Thus, Nox1 seems to be important for the progression of diabetes-associated atherosclerosis while in atherosclerosis there are inconsistent data as to whether Nox1 is protective and further research is needed.

Nox2 (previously called gp91) deletion in ApoE^-/-^ mice on a normal chow diet does not seem to impact on lesion area and lipid deposition in the aortic sinus at the age of 24 weeks with male ApoE^-/-^/gp91^-/-^ mice having decreased HDL levels and increased LDL levels by 60% compared to ApoE^-/-^ whereas these differences in plasma cholesterol were not evident in female double knockout mice [233]. ApoE^-/-^ mice overexpressing Nox2 specifically in the endothelial cells (Nox2Tg ApoE^-/-^ mice) on a normal chow diet have elevated vascular superoxide production, but surprisingly, the lesion area is equivalent to ApoE^-/-^ mice with comparable lesion composition and progression [225]. Interestingly, VCAM1 expression–an inflammatory marker of endothelial cells involved in the development of atherosclerosis–and macrophage recruitment are both increased at the age of 9 weeks to similar levels seen in ApoE^-/-^ mice [225]. Therefore, it has been suggested that Nox2 plays a significant role to the initiation of lesion formation and not of lesion progression [225]. On the other hand, Nox2^-/y^/ApoE^-/-^ male mice administered a western-type diet (21% w/w fat, 0.15% cholesterol) either for 7 or 14 weeks have similar plasma cholesterol levels with ApoE^-/-^ and although the lesion area coverage in the aortic sinus is similar, a 50% decrease in lesion area throughout the aorta is evident [57]. Furthermore, Nox2^-/y^/ApoE^-/-^ exhibit reduced vascular superoxide production by 75% and improved nitric oxide bioavailability compared to ApoE^-/-^ [57]. Importantly, ApoE^-/-^ mice on a western-type diet (15.8% w/w fat, 1.25% cholesterol) and treated with a Nox2-specific inhibitor, the Nox2ds-tat peptide, showed decreased lesion coverage throughout the aorta and the carotid arteries [94]. Moreover, vascular superoxide production and mRNA expression of p47phox and p22phox were decreased in the carotid arteries [94]. The Nox2ds-tat peptide is a 9 amino acid sequence (similar to the cytosolic B-loop of Nox2) and inhibits the interaction between Nox2 and p47phox. Nox2ds-tat suppresses superoxide production in endothelial cells and its efficacy has been established both in vitro and in vivo [234–236]. This evidence suggests that Nox2 may be an important contributor in the initiation of atherogenesis and its role in the progression of atherosclerosis remains to be further elucidated [57, 94, 225].

Skeletal muscle expresses two Nox members i.e. Nox2 and Nox4 although in cultured muscle cells apart from Nox2 and 4 expression, Nox1 and Duox 1 and 2 expression has been also reported [237]. Nox2 is localised in the sarcolemma, transverse tubules and possibly in the sarcoplasmic reticulum whereas Nox4 is localised in mitochondria and sarcoplasmic reticulum [138, 237, 238]. Noxs along with mitochondria are considered to be the major sources of ROS production in the skeletal muscle [138]. In particular, Nox2 is an important source of ROS production in response to skeletal muscle contraction and mechanotransduction as well as in insulin signalling [237]. In several chronic cardiac and skeletal muscle diseases, such heart failure and ageing-induced sarcopenia there is an upregulation of Nox2 subunits in the muscle and recently Nox2 was recognised as a major contributor to the pathology of Duchenne Muscular Dystrophy [237]. Sparse evidence also suggests a role for Nox2 in insulin resistance in the muscle [237]. An increase in ROS production and Nox2 protein expression by 1.6-fold and p47phox subunit by 7-fold has been found in muscle of insulin resistant C57Bl/6 mice caused by high-fat feeding for 8 weeks [91]. Nox2 knockout mice on a high-fat diet (60% kcal from fat) for 9 months showed improved glucose uptake and reduced ROS production compared with wild type mice [239]. Consequently, Noxs and especially Nox2 play an important role in muscle oxidative stress both in physiological and pathophysiological conditions. Exciting recent research is focusing on the pharmacological inhibition of Noxs by small molecule inhibitors for counteracting the atherothrombotic process [240–243]. However, most animal studies employing small molecule inhibitors such as the Vaso-pharm triazolo pyrimidine derivatives, apocynin and diphenyleneiodonium either lack specificity or have adverse effects [241, 244, 245]. Recently ebselen and its analogues were identified as specific inhibitors of Nox2 and of Nox1, with the JM-77b derivative having a greater specificity for Nox2 than Nox1, Nox4 and Nox5 [240–242]. Similarly, GKT136901 and GKT137831, a pyrazolopyridine class of compounds, are specific inhibitors of both Nox1 and Nox4. Administration of the GKT137831 inhibitor has been shown to reduce liver fibrosis and diabetic atherosclerosis in animal studies [232, 241]. It is worth mentioning that such compounds are the first oral inhibitors that are in phase II clinical trials for treatment of diabetic nephropathy [241, 246]. However, selective inhibition of the Nox homologues is essential in order to eliminate opposing effects and complications in cardiovascular pathologies [26, 241, 247, 248]. Collectively, although small molecule inhibitors are a promising new area, further validation of their pharmacokinetic and pharmacodynamic profile is of paramount significance so as to proceed in clinical trials [241].

## Atherosclerosis and skeletal muscle deficits; possible links through vasculature, ischemia and inflammation

The “metabolic hypothesis” was introduced almost three decades ago to suggest a direct relationship between the local blood flow and the metabolic demand of the muscle [249, 250]. The crosstalk between blood flow and tissue metabolism is brought about by products of ATP turnover released by the muscle, in order to regulate the vascular tone. More recently NO, prostaglandins and endothelium-derived hyperpolarization factors secreted by endothelial cells were also included as potent regulators of vascular tone and perfusion in the skeletal muscle [251–254]. Apart from the nutrient and oxygen supply, the interplay between the vasculature and the skeletal muscle is evident in satellite cell regulation. Satellite cells are in close proximity with capillaries and their number correlates with the number of capillaries per myofibre [255]. In vitro studies have shown that growth factors, such as insulin-like growth factor (IGF-1), hepatocyte growth factor and basic fibroblast growth factor, derived from endothelial cells promote the activation of satellite cells in a paracrine way and in turn satellite cells have proangiogenic activity mainly through the secretion of VEGF [256, 257]. Similarly, angiopoietin 1 that is expressed by satellite cells, smooth muscle cells and pericytes may contribute not only to vessel stability but also to satellite cell quiescence [255]. Additional evidence suggests that skeletal muscle acts as a secretory organ and cross talks to vasculature [123, 258]. Several cytokines, such as IL-6, IL-10, IL-15, IL-7 as well as other factors such as myostatin and follistatin are secreted by muscle cells and act upon cells in both an autocrine and paracrine way [258, 259]. It was recently shown that the endothelium contributes to muscle regeneration after eccentric exercise by expressing leukemia inhibitor factor (LIF), fraktalkine, VEGF and IL-8 that contribute to immune cell infiltration and angiogenesis, two essential processes for muscle regeneration [259]. Myokines–defined as those factors secreted by the skeletal muscle per se–have been speculated to exhibit an endocrine function affecting other organs such as the liver and adipose tissue, although this line of thought remains to be further established [258, 259].

Most studies that investigate the impact of atherosclerosis on PAD focus on haemodynamic changes from ischemia-reperfusion cycles [14, 144, 260–264]. Skeletal muscle pathophysiology of ischemia-reperfusion cycles has been recently reviewed in detail amalgamating data from both human and animal studies of PAD [134]. In particular, chronic ischemia causes deprivation of oxygen and nutrient supply, leading to several changes in skeletal muscle metabolism. Such changes include: i) decrease in mitochondrial respiration, ii) increase in ROS production, iii) accumulation of products of metabolism such as H^+^ and Ca^+^ and iv) dysregulation of myocellular ions, (i.e. K^+^ and Na^+^) that further compromise cellular function [134]. The onset of reperfusion has also a negative impact on the ischemic muscle mainly due to a rapid increase in oxygen supply [134]. Higher amounts of oxygen trigger overproduction of ROS by endothelial cells, pro-inflammatory cells and by myocytes with the latter producing ROS mainly due to uncoupling of mitochondrial respiration [134]. Furthermore, satellite cells in the hindlimb ischemia model seem to have decreased expression of myogenic markers of differentiation which is restored once reperfusion is established [265]. This finding further highlights the interaction between the vasculature and the skeletal muscle.

There is currently sparse experimental evidence on the effect of central atherosclerosis on skeletal muscle biology. Hyperlipidaemia–a major risk factor of atherosclerosis and PAD–is considered an important step in atherogenesis [23, 266–268]. Excess cholesterol leads to endothelial dysfunction that in turn promotes expression of pro-inflammatory cytokines, such as TNFα and IL-6 and ROS overproduction [23, 266]. ROS production from several enzymes such as NADPH oxidases, xanthine oxidase and uncoupled eNOS lead to oxidative stress and activation of NF-κB, which enhances the expression of pro-inflammatory cytokines, such as TNFα and IL-6 and reinforces further production of ROS [269–272]. In addition, pro-inflammatory cytokines promote further activation of redox-sensitive transcription factors by means of several signalling pathways that contribute to additional ROS production from the vasculature [273, 274].

Since hyperlipidaemia impacts on the liver and adipose tissue, it can be speculated that skeletal muscle can be affected as well taken into account the molecular crosstalk among these metabolically active tissues [147, 275–278]. In fact, triglyceride accumulation in the adipose tissue leads to increased expression of pro-inflammatory cytokines and adipokines, such as resistin and visfatin that beyond promoting atherosclerosis, could potentially induce oxidative stress in skeletal muscle [147, 276–279]. Systemic low grade inflammation is evident in both Ldlr^-/-^ and ApoE^-/-^ experimental atherosclerotic mouse models with increased plasma concentrations of TNFα, IL1β and MCP1 [194]. Pro-inflammatory cytokines in circulation could trigger redox-sensitive transcription factors in skeletal muscle promoting ROS and pro-inflammatory cytokine production from muscle cells in a similar way as in insulin resistant patients [277, 278]. Collectively, it can be speculated that systemic low grade inflammation may also affect skeletal muscle metabolic homeostasis in PAD.

Another factor that may be affecting muscle pathophysiology is the peptide hormone angiotensin II that has been found to contribute to atherogenesis and progression of atherosclerosis [280]. Angiotensin II is a potent vasoconstrictor and pro-inflammatory peptide that induces vascular endothelial dysfunction in multiple ways by enhancing i) the secretion of pro-inflammatory cytokines, ii) production of ROS, iii) expression of oxidised LDL receptor and iv) expression of matrix metalloproteinases from the endothelium [280]. Moreover, angiotensin II and its receptors are expressed in human atherosclerotic plaques from the majority of the cells that reside in the plaque such as macrophages, endothelial cells and smooth muscle cells [280]. Similarly, angiotensin II and its receptor angiotensin II type-1 receptor (AT1) are expressed in lesions of atherosclerotic animal models [280]. The substantial role of angiotensin II in atherosclerosis progression has been demonstrated in AT1^-/-^/ApoE^-/-^ mice that exhibited diminished atherosclerotic burden and vascular ROS production [281]. Importantly, treatment of ApoE^-/-^ mice with an AT1 receptor antagonist inhibited progression of atherosclerosis [281]. In a different study, treatment of ApoE^-/-^ mice with an AT1 receptor antagonist and simultaneous infusion with angiotensin II inhibited progression of atherosclerosis by decreasing the expression of pro-inflammatory markers and ROS production from the endothelium [282]. The aforementioned effects of angiotensin II on progression of atherosclerosis are independent of systemic blood pressure changes, since treatment of ApoE^-/-^ mice with a calcium antagonist or a vasodilator did not affect lesion progression [281, 282].

Angiotensin II might affect skeletal muscle homeostasis in atherosclerosis, not only due to its significant impact on the vasculature, but due to a rather direct effect on skeletal muscle. For instance, angiotensin II in congestive heart failure and chronic kidney disease contributes to muscle wasting [283, 284]. This may be explained by a downregulation of the Akt/mTOR/p70S6K pathway and activation of apoptotic pathways (e.g. activation of caspase 3, proteins ubiquitination, reduction of Bad phosphorylation and increase in cytosolic cytochrome c) [283]. Specific overexpression of IGF-1 in skeletal muscle attenuated the effects of angiotensin II and it was concluded that downregulation of IGF-1 may be the primary cause of muscle wasting in congestive heart failure and chronic kidney disease [283]. More recently angiotensin II was shown to cause mitochondrial dysfunction by suppressing AMPK phosphorylation through protein phosphatase 2C-alpha and thus reducing mitochondrial biogenesis, activity of complex IV, expression of complex V and inhibiting mitophagy [284]. In addition, mitochondrial dysfunction from angiotensin II was also induced through AMPK independent pathways mainly by inhibition of proteins that are associated with mitochondrial fission and fusion [284]. Notably, angiotensin II may increase oxidative stress in skeletal muscle by inducing Noxs and reduces satellite cell-driven muscle regeneration [237, 285, 286]. Angiotensin II, in particular, inhibits Notch signalling and cyclins D1 and E in satellite cells, thus compromising the satellite cell proliferation and differentiation potency [286]. Consequently, angiotensin II may potentially affect skeletal muscle physiology in the context of PAD, although the mechanistic links between atherosclerosis and skeletal muscle remain to be discovered.

## Conclusions

Peripheral atherosclerosis has serious implications for human health and impacts in several tissues including skeletal muscle. Accumulating data over the past decade from human studies with PAD patients and some evidence from rodent studies have improved our understanding about the pathophysiology of skeletal muscle in the context of atherosclerosis. There is a number of structural, functional, biochemical and metabolic deficits taking place in the skeletal muscle that compromise its homeostasis and functional capacity. There are signs of myopathy, fibrosis, mitochondrial de-regulation, oxidative stress, inflammation and apoptosis, followed by impaired capillary density, chronic ischemia and muscle pain. Such manifestations in skeletal muscle may in turn compromise the capacity to generate force or ambulate and impact on the quality of life. By identifying the crossroads between skeletal muscle metabolism, dietary challenges and peripheral atherosclerosis we will be able to better prepare new strategies to cope with such abnormalities and improve muscle physiology and quality of life in patients with cardiovascular complications such as peripheral atherosclerosis. Experimental models of atherosclerosis with a skeletal muscle phenotype may offer an attractive tool to develop new strategies to combat the manifestations of atherosclerosis in peripheral tissues. NADPH oxidases are key components of increased cellular oxidative stress and their role in atherogenesis has received much attention recently. Several studies have shown the beneficial effect of Nox2 deficiency in the initiation and lesion progression in the aorta of the ApoE^-/-^ mice. Several inhibitors have been developed in an attempt to reduce atherosclerosis, with Nox2ds-tat recently showing that treatment of ApoE^-/-^ with this peptide reduced lesion size in the aorta and carotid arteries [94]. The impact of Nox inhibition on skeletal muscle and whole body levels of oxidative stress in the context of atherosclerosis or other cardiovascular disorders remains to be established.

## Abbreviations

4-HNE: 4-hydroxy-2-nonenal
ABI: Ankle-brachial index
*ADAR*: Double-stranded RNA-specific adenosine deaminase
Akt: Protein kinase B
AngII: Angiotensin II
APOB: Apolipoprotein B
ApoE: Apolipoprotein E
ApoE^-/-^: Apolipoprotein E knockout
ApoE^-/-^/gp91^-/-^: Double Apolipoprotein E knockout and gp91 knockout
ApoE^-/-^/p47^-/-^: Double Apolipoprotein E knockout and p47phox knockout
APOE2: Apolipoprotein E isoform 2
APOE3: Apolipoprotein E isoform 3
APOE4: Apolipoprotein E isoform 4
AT: Adipose tissue
AT1 receptor: Angiotensin II receptor type 1
BMO: Bone marrow transplantation from ApoE^-/-^/p47phox^-/-^ to ApoE^-/-^
BW: Body weight
Ca^+^: Calcium ion
CAD: Coronary artery disease
*CHORD1*: Cysteine and histidine rich domain containing 1
CLI: Critical limb ischemia
*COL1A1*: Collagen Type I Alpha 1 Chain
*COL3A1*: Collagen Type III Alpha 1 Chain
CSA: Cross sectional area
*CTSB*: Cathepsin B
*CTSC*: Cathepsin C
DHE: Dihydroethidium
DUOX1 and 2: Dual Oxidase 1 and 2
eNOS: Endothelial nitric oxide synthase
ET-1: Endothelin 1
ETC: Electron transport chain complexes
FoxO1: Forkhead Box Protein O1
FoxO3: Forkhead Box Protein O3
*GCS*: Glycine cleavage system protein H
Glut4: Glucose Transporter Type 4
gp91^-/-^: gp91 knockout
gp91phox: Superoxide-Generating NADPH Oxidase Heavy Chain Subunit
GSH: Glutathione reduced
GSSG: glutathione oxidised
GT test: Graded treadmill test
GTPases: Guanosine triphosphatases
H^+^: Hydrogen ion/proton
H_2_O_2_: Hydrogen peroxide
HDL: High-density lipoprotein
HFD: High-fat Diet
HIF1α: Hypoxia inducible factor 1α
HOMA-IR: Homeostatic model assessment for insulin resistance
IC: Intermittent claudication
iCAM-1: Intercellular adhesion molecule 1
IDL: Intermediate density lipoproteins
*IFI30*: Gamma-interferon-inducible lysosomal thiol reductase
IGF-1: Insulin-like growth factor 1
IL-10: Interleukin 10
IL-15: Interleukin 15
IL-1β: Interleukin 1 beta
IL-6: Interleukin 6
IL-7: Interleukin 7
K^+^: Potassium ion
LDL: Low-density lipoprotein
LDLR: Low-density lipoprotein receptor
LDLR^-/-^: Low-density lipoprotein receptor knockout
*MARCKS*: Myristoylated alanine-rich C kinase substrate
MCP1: Monocyte chemotactic protein 1
MMP9: Matrix metalloproteinase 9
*MPPED2*: Metallophosphoesterase domain containing 2
Na^+^: Sodium ion
NADPH: Nicotinamide adenine dinucleotide phosphate
ND: Normal diet (chow diet)
*NDD9*: Neural precursor cell expressed developmentally down-regulated protein 9
NF-κB: Nuclear factor kappa-light-chain-enhancer of activated B cells
NO: Nitric oxide
Nox1: Nicotinamide adenine dinucleotide phosphate oxidase 1
Nox1^-/y^/ApoE^-/-^: Double Apolipoprotein E knockout and Nox1 knockout
Nox2: Nicotinamide adenine dinucleotide phosphate oxidase 2
Nox2^-/y^: Nox2 Knockout
Nox2^-/y^/ApoE^-/-^: Double Apolipoprotein E knockout and Nox2 knockout
Nox2Tg ApoE^-/-^: ApoE^-/-^ with overexpression of Nox2 specifically in Endothelial Cells
Nox3: Nicotinamide adenine dinucleotide phosphate oxidase 3
Nox4: Nicotinamide adenine dinucleotide phosphate oxidase 4
Nox5: Nicotinamide adenine dinucleotide phosphate oxidase 5
Noxa1: NADPH Oxidase Activator 1
Noxo1: NADPH Oxidase Organiser 1
Noxs: Nicotinamide adenine dinucleotide phosphate oxidases
NS: Not specified
O_2_^.-^: Superoxide
oxLDL: Oxidised LDL
p22phox: Superoxide-Generating NADPH Oxidase Light Chain Subunit
p27^KIP1^: Cyclin dependent kinase inhibitor 1B
p40phox: Neutrophil cytosol factor 4
p47phox: Neutrophil cytosol factor 1
p47phox^-/-^: p47phox knockout
p67phox: NADPH oxidase activator 2
PAD: Peripheral arterial disease
*PLA24A*: Phospholipase A2 group IVA
*PLP*: Phospholipid transfer protein
Rac1: Ras-related C3 botulinum toxin substrate 1
Rac2: Ras-related C3 botulinum toxin substrate 2
ROS: Reactive oxygen species
shRNA Nox2-expressing: Short Hairpin RNA to silence Nox2 expression
SMC: Smooth muscle cells
SOD: Superoxide Dismutase
SOD2: superoxide dismutase 2
TBARS: Thiobarbituric acid reactive substances
TGs: Triglycerides
Th1: T-helper Type 1 cells
Th17: T-helper Type 17 cells
Th2: T-helper Type 2 cells
THSB1: Thrombospondin 1
TNFα: Tumour necrosis factor α
*TREM1*: Triggering receptor expressed on myeloid cells 1
VCAM1: Vascular cell adhesion molecule 1 expression
VEGF: Vascular endothelial growth factor
VEGF165b: Vascular endothelial growth factor 165b
VEGF-A: Vascular endothelial growth factor A
VLDL: Very low-density lipoprotein
VO_2max_: Maximal oxygen consumption
VSMC: vascular smooth muscle cells
VWO: Bone Marrow transplantation from ApoE^-/-^ to ApoE^-/-^/p47phox^-/-^
WD: Western-type diet
WT: Wild type
